# Functional Genomics and Immunologic Tools: The Impact of Viral and Host Genetic Variations on the Outcome of Zika Virus Infection

**DOI:** 10.3390/v10080422

**Published:** 2018-08-11

**Authors:** Sang-Im Yun, Byung-Hak Song, Jordan C. Frank, Justin G. Julander, Aaron L. Olsen, Irina A. Polejaeva, Christopher J. Davies, Kenneth L. White, Young-Min Lee

**Affiliations:** 1 Department of Animal Dairy and Veterinary Sciences, College of Agriculture and Applied Sciences, Utah State University, Logan, UT 84322, USA; sangim.yun@usu.edu (S.-I.Y.); byunghak.song@aggiemail.usu.edu (B.-H.S.); jc.frank@aggiemail.usu.edu (J.C.F); justin.julander@usu.edu (J.G.J); aaron.olsen@usu.edu (A.L.O.); irina.polejaeva@usu.edu (I.A.P.); chris.davies@usu.edu (C.J.D.); ken.white@usu.edu (K.L.W.); 2 Institute for Antiviral Research, Utah State University, Logan, UT 84322, USA; 3 Veterinary Diagnostics and Infectious Diseases, Utah Science Technology and Research, Utah State University, Logan, UT 84341, USA

**Keywords:** Zika virus, flavivirus, infectious cDNA, replication, gene expression, neuropathogenesis, viral genetic variation, host genetic variation

## Abstract

Zika virus (ZIKV) causes no-to-mild symptoms or severe neurological disorders. To investigate the importance of viral and host genetic variations in determining ZIKV infection outcomes, we created three full-length infectious cDNA clones as bacterial artificial chromosomes for each of three spatiotemporally distinct and genetically divergent ZIKVs: MR-766 (Uganda, 1947), P6-740 (Malaysia, 1966), and PRVABC-59 (Puerto Rico, 2015). Using the three molecularly cloned ZIKVs, together with 13 ZIKV region-specific polyclonal antibodies covering nearly the entire viral protein-coding region, we made three conceptual advances: (i) We created a comprehensive genome-wide portrait of ZIKV gene products and their related species, with several previously undescribed gene products identified in the case of all three molecularly cloned ZIKVs. (ii) We found that ZIKV has a broad cell tropism in vitro, being capable of establishing productive infection in 16 of 17 animal cell lines from 12 different species, although its growth kinetics varied depending on both the specific virus strain and host cell line. More importantly, we identified one ZIKV-non-susceptible bovine cell line that has a block in viral entry but fully supports the subsequent post-entry steps. (iii) We showed that in mice, the three molecularly cloned ZIKVs differ in their neuropathogenicity, depending on the particular combination of viral and host genetic backgrounds, as well as in the presence or absence of type I/II interferon signaling. Overall, our findings demonstrate the impact of viral and host genetic variations on the replication kinetics and neuropathogenicity of ZIKV and provide multiple avenues for developing and testing medical countermeasures against ZIKV.

## 1. Introduction

Discovered in Uganda in 1947 in a febrile rhesus macaque [1], Zika virus (ZIKV) is a medically important flavivirus [2] related to Japanese encephalitis (JEV), West Nile (WNV), dengue, and yellow fever viruses [3]. Originally, it was confined within an equatorial belt running from Africa to Asia, with only about a dozen cases of human illness reported [4]. In 2007, however, it caused a major outbreak of mild illness characterized by fever, rash, arthralgia, and conjunctivitis on the western Pacific Island of Yap [5,6]. Since then, it has spread eastward across the Pacific Ocean, invading French Polynesia and other Pacific Islands in 2013–2014 [7], reaching the Americas and Caribbean in 2015–2016 [8,9], and now threatening much of the world [10,11]. ZIKV is spread to humans mainly through the bite of an infected *Aedes* species mosquito, e.g., *Aedes aegypti* or *Aedes albopictus* [12], but it can also be transmitted from a mother to her child during pregnancy [13,14] or through sexual contact [15,16]. Serious concerns have been raised over links to congenital neurological malformations (e.g., microcephaly) and severe neurological complications (e.g., Guillain–Barré syndrome) [17,18]. Despite its continuous rapid spread and high pandemic potential, no vaccine or drug is available to prevent or treat ZIKV infection.

ZIKV is an enveloped RNA virus with a nucleocapsid core comprising an ~11 kb plus-strand RNA genome and multiple copies of the C protein; this core is surrounded by a lipid bilayer bearing the anchored M and E proteins [19,20]. With regard to the molecular events that occur during ZIKV infection, our current understanding of the molecular biology of closely related flaviviruses offers a promising starting point for ZIKV research [21]. As the first step in flavivirus replication, the virion binds to one or more cellular proteins on the surface of a host cell, and is then internalized via clathrin-mediated endocytosis in a viral glycoprotein E-dependent manner [22,23,24]. Within endosomes, the E glycoprotein undergoes low pH-induced conformational changes, followed by fusion of the viral and host cell membranes [25,26,27]. In the cytoplasm, the viral genomic RNA functions initially as an mRNA for the translation of a single long open reading frame (ORF) flanked by 5′ and 3′ non-coding regions (NCRs) [28,29]; the resulting polyprotein is cleaved by viral and cellular proteases to generate at least 10 mature proteins [30,31]: three structural (C, prM, and E) and seven nonstructural (NS1, 2A, 2B, 3, 4A, 4B, and 5). In JEV and WNV, ribosomal frameshifting is also used for the expression of NS1’, a C-terminally extended form of NS1 [32,33,34]. A complex of the seven nonstructural proteins directs viral RNA replication on the distinct virus-induced membranous compartments derived from endoplasmic reticulum (ER) [35,36]. This replication process is catalyzed by two main viral components: (i) NS3, with serine protease (and its cofactor, NS2B) and RNA helicase/NTPase/RTPase activity, and (ii) NS5, with methyltransferase/guanylyltransferase and RNA-dependent RNA polymerase activity [37]. Virus assembly begins with budding of the C proteins, complexed with a newly made viral genomic RNA, into the ER lumen, and acquisition of the viral prM and E proteins. The prM-containing immature virions travel through the secretory pathway; in the trans-Golgi network, a cellular furin-like protease cleaves prM to yield the mature M protein, converting the immature particle to a mature virion [38].

The clinical presentation of ZIKV infection is highly variable, ranging from no apparent symptoms or mild self-limiting illness, to severe neurological disorders, such as microcephaly and Guillain–Barré syndrome [10,17]. Fundamentally, the varied outcomes after infection with a pathogen depend on the specific combination of pathogen and host genotypes [39]. On the virus side, a limited but significant number of ZIKVs have been isolated from Africa, Asia, and the Americas during the past 70 years. Recent phylogenetic analyses based on complete or near-complete viral genome sequences have revealed that the spatiotemporally distinct ZIKV strains are grouped into two major genetic lineages, African and Asian, with the 2015–2016 American epidemic strains originating from a common ancestor of the Asian lineage [40,41,42]. Despite the continuous expansion of its genetic diversity, little is known about the effect of viral genetic variation on the pathogenicity of ZIKV between the two lineages or between different strains within a particular lineage. On the host side, much progress has recently been made in developing murine models for ZIKV infection [43], including mice genetically engineered to lack one or more components of the innate and adaptive immune systems that affect the development, severity, and progression of ZIKV-induced disease [44,45,46,47,48,49]. However, the influence of host genetic variation on susceptibility to ZIKV infection is largely unknown.

To assess, experimentally, the impact of viral and host genetic variations on the outcome of ZIKV infection, we have now generated (i) a unique panel of three functional bacterial artificial chromosomes (BACs), each containing a full-length infectious cDNA for one of three genetically divergent ZIKV strains, and (ii) an exclusive collection of 13 rabbit antisera capable of detecting almost all of the ZIKV gene products and their related species. Using these functional genomics and immunologic tools, together with various cell culture and mouse infection model systems, we show that the three molecularly defined cDNA-derived ZIKVs have a similar viral protein expression profile, but display biologically significant differences in in vitro growth properties and in vivo neuropathogenic potential that depend on both viral and host genetic traits. Our study not only provides a powerful system for the functional study of viral and host genetics in ZIKV replication and pathogenesis, but also offers a valuable platform for the rational design of vaccines and therapeutics against ZIKV.

## 2. Materials and Methods

### 2.1. Cells and Viruses

Details of the 17 cell lines used in this study, including their growth medium and culture conditions, are presented in Table 1. ZIKV MR-766 and P6-740 were obtained from the World Reference Center for Emerging Viruses and Arboviruses, University of Texas Medical Branch (Galveston, TX, USA), and ZIKV PRVABC-59 was provided by the Centers for Disease Control and Prevention (Fort Collins, CO, USA). In the case of all three ZIKVs, viral stocks were amplified once in Vero cells at a multiplicity of infection (MOI) of 1.

### 2.2. Sequence Alignment and Phylogenetic Analysis

Multiple sequence alignments were performed via ClustalX, and the phylogenetic tree was constructed using MEGA and visualized via TreeView, as described [50]. Sequence identities between aligned nucleotide and amino acid sequences were calculated using ClustalX.

### 2.3. Cloning

Standard molecular cloning techniques were used to create three full-length ZIKV cDNAs [51], one each for MR-766, P6-740, and PRVABC-59 in the BAC plasmid pBeloBAC11 [52], designated pBac/MR-766, pBac/P6-740, and pBac/PRVABC-59. The same cloning strategy was used to construct all three full-length ZIKV cDNAs with the appropriate primer sets listed in Table 2. Essentially, each full-length ZIKV cDNA flanked by the 5′ SP6 promoter and the 3′ *Psr*I/*Bar*I restriction enzyme site was created by joining five overlapping RT-PCR-generated cDNA fragments at four natural restriction enzyme sites found in the viral genome (see below for detailed description of cloning strategy). The cloned cDNAs were checked by restriction enzyme mapping and sequencing.

(1) pBac/MR-766: The genomic RNA of ZIKV MR-766 (GenBank accession no. KX377335) was used as a template for the synthesis of three overlapping cDNA fragments by RT-PCR with the following primer sets: Frag-A^MR-766^ (4552 bp), Z1RT, and Z1F + Z1R; Frag-B^MR-766^ (5070 bp), Z2RT, and Z2F + Z2R; and Frag-C^MR-766^ (5008 bp), Z3RT, and Z3F + Z3R. Each of the three cDNA amplicons was subcloned into pBAC^SP6^/JVFLx/XbaI [53], a derivative of the pBeloBAC11 plasmid, by ligating the 8381 bp *Pme*I-*Mlu*I fragment of pBAC^SP6^/JVFLx/XbaI with the 4538, 5056, and 4994 bp *Pme*I-*Asc*I fragments of the Frag-A^MR-766^, Frag-B^MR-766^, and Frag-C^MR-766^ amplicons, respectively. This generated pBac/Frag-A^MR-766^ to -C^MR-766^. To introduce an SP6 promoter immediately upstream of the first adenine residue of the viral genome, two cDNA fragments were first amplified individually by (i) PCR of pBAC^SP6^/JVFLx/XbaI with a pair of primers, S123-5sp1F + S123-5sp1R (S123-5sp1R contains the antisense sequence of the SP6 promoter) and (ii) PCR of pRs/5′NCR^MR-766^ [54] with another pair of primers, S1-5sp2F + S1-5sp2R. Subsequently, these two fragments were fused by a second round of PCR with the outer forward and reverse primers S123-5sp1F + S1-5sp2R. The 1025 bp *Bam*HI-*Sac*II fragment of the fused PCR amplicons was ligated with the 2718 bp *Bam*HI-*Sac*II fragment of pRs2, creating pRs/5′SP^MR-766^. To engineer a unique *Psr*I run-off site just downstream of the last thymine residue of the viral genome, one cDNA fragment was amplified by PCR of pRs/3′NCR^MR-766^ [54] with primers S1-3roF + S1-3roR (S1-3roR contains the antisense sequence of the *Psr*I and *Not*I recognition sites in a row). The 649 bp *Sac*II-*Not*I fragment of the resulting amplicons was ligated with the 2667 bp *Sac*II-*Not*I fragment of pRs2, creating pRs/3′RO^MR-766^. The full-length MR-766 cDNA clone pBac/MR-766 was then assembled by sequentially joining the 7456 bp *Pac*I-*Not*I fragment of pBAC^SP6^/JVFLx/XbaI with the following five DNA fragments: (i) the 1004 bp *Pac*I-*Xma*I fragment of pRs/5’SP^MR-766^, (ii) the 3160 bp *Xma*I-*Xho*I fragment of pBac/Frag-A^MR-766^, (iii) the 3144 bp *Xho*I-*Nsi*I fragment of pBac/Frag-B^MR-766^, (iv) the 3041 bp *Nsi*I-*Bam*HI fragment of pBac/Frag-C^MR-766^, and (v) the 619 bp *Bam*HI-*Not*I fragment of pRs/3′RO^MR-766^.

(2) pBac/P6-740: The genomic RNA of ZIKV P6-740 (GenBank accession no. KX377336) was used as a template for the synthesis of three overlapping cDNA fragments by RT-PCR with the following primer sets: Frag-A^P6-740^ (4553 bp), Z1RT, and Z1F + Z1R; Frag-B^P6-740^ (5070 bp), Z2RT, and Z2F + Z2R; and Frag-C^P6-740^ (5008 bp), Z3RT, and Z3F + Z3R. Each of the three cDNA amplicons was subcloned into pBAC^SP6^/JVFLx/XbaI, by ligating the 8381 bp *Pme*I-*Mlu*I fragment of pBAC^SP6^/JVFLx/XbaI with the 4539, 5056, and 4994 bp *Pme*I-*Asc*I fragments of the Frag-A^P6-740^, Frag-B^P6-740^, and Frag-C^P6-740^ amplicons, respectively. This generated pBac/Frag-A^P6-740^ to -C^P6-740^. To introduce an SP6 promoter immediately upstream of the first adenine residue of the viral genome, two cDNA fragments were first amplified individually by (i) PCR of pBAC^SP6^/JVFLx/XbaI with a pair of primers, S123-5sp1F + S123-5sp1R (S123-5sp1R contains the antisense sequence of the SP6 promoter) and (ii) PCR of pRs/5′NCR^P6-740^ [54] with another pair of primers, S23-5sp2F + S23-5sp2R. Subsequently, these two fragments were fused by a second round of PCR with the outer forward and reverse primers S123-5sp1F + S23-5sp2R. The 1025 bp *Bam*HI-*Sac*II fragment of the fused PCR amplicons was ligated with the 2718 bp *Bam*HI-*Sac*II fragment of pRs2, creating pRs/5′SP^P6-740^. To engineer a unique *Bar*I run-off site just downstream of the last thymine residue of the viral genome, one cDNA fragment was amplified by PCR of pRs/3′NCR^P6-740^ [54] with primers S23-3roF + S23-3roR (S23-3roR contains the antisense sequence of the *Bar*I and *Not*I recognition sites in a row). The 649 bp *Sac*II-*Not*I fragment of the resulting amplicons was ligated with the 2667 bp *Sac*II-*Not*I fragment of pRs2, creating pRs/3′RO^P6-740^. The full-length P6-740 cDNA clone pBac/P6-740 was then assembled by sequentially joining the 7456 bp *Pac*I-*Not*I fragment of pBAC^SP6^/JVFLx/XbaI with the following five DNA fragments: (i) the 187 bp *Pac*I-*Nhe*I fragment of pRs/5′SP^P6-740^, (ii) the 2930 bp *Nhe*I-*Spe*I fragment of pBac/Frag-A^P6-740^, (iii) the 3359 bp *Spe*I-*Ngo*MIV fragment of pBac/Frag-B^P6-740^, (iv) the 4059 bp *Ngo*MIV-*Stu*I fragment of pBac/Frag-C^P6-740^, and (v) the 433 bp *Stu*I-*Not*I fragment of pRs/3′RO^P6-740^.

(3) pBac/PRVABC-59: The genomic RNA of ZIKV PRVABC-59 (GenBank accession no. KX377337) was used as a template for the synthesis of three overlapping cDNA fragments by RT-PCR with the following primer sets: Frag-A^PRVABC-59^ (4553 bp), Z1RT, and Z1F + Z1R; Frag-B^PRVABC-59^ (5070 bp), Z2RT, and Z2F + Z2R; and Frag-C^PRVABC-59^ (5008 bp), Z3RT, and Z3F + Z3R. Each of the three cDNA amplicons was subcloned into pBAC^SP6^/JVFLx/XbaI, by ligating the 8381 bp *Pme*I-*Mlu*I fragment of pBAC^SP6^/JVFLx/XbaI with the 4539, 5056, and 4994 bp *Pme*I-*Asc*I fragments of the Frag-A^PRVABC-59^, Frag-B^PRVABC-59^, and Frag-C^PRVABC-59^ amplicons, respectively. This generated pBac/Frag-A^PRVABC-59^ to -C^PRVABC-59^. To introduce an SP6 promoter immediately upstream of the first adenine residue of the viral genome, two cDNA fragments were first amplified individually by (i) PCR of pBAC^SP6^/JVFLx/XbaI with a pair of primers, S123-5sp1F + S123-5sp1R (S123-5sp1R contains the antisense sequence of the SP6 promoter) and (ii) PCR of pRs/5′NCR^PRVABC-59^ [54] with another pair of primers, S23-5sp2F + S23-5sp2R. Subsequently, these two fragments were fused by a second round of PCR with the outer forward and reverse primers S123-5sp1F + S23-5sp2R. The 1025 bp *Bam*HI-*Sac*II fragment of the fused PCR amplicons was ligated with the 2718 bp *Bam*HI-*Sac*II fragment of pRs2, creating pRs/5′SP^PRVABC-59^. To engineer a unique *Bar*I run-off site just downstream of the last thymine residue of the viral genome, one cDNA fragment was amplified by PCR of pRs/3′NCR^PRVABC-59^ [54] with primers S23-3roF + S23-3roR (S23-3roR contains the antisense sequence of the *Bar*I and *Not*I recognition sites in a row). The 649 bp *Sac*II-*Not*I fragment of the resulting amplicons was ligated with the 2667 bp *Sac*II-*Not*I fragment of pRs2, creating pRs/3′RO^PRVABC-59^. The full-length PRVABC-59 cDNA clone pBac/PRVABC-59 was then assembled by sequentially joining the 7456 bp *Pac*I-*Not*I fragment of pBAC^SP6^/JVFLx/XbaI with the following five DNA fragments: (i) the 187 bp *Pac*I-*Nhe*I fragment of pRs/5′SP^PRVABC-59^, (ii) the 4426 bp *Nhe*I-*Eco*NI fragment of pBac/Frag-A^PRVABC-59^, (iii) the 2114 bp *Eco*NI-*Sac*II fragment of pBac/Frag-B^PRVABC-59^, (iv) the 3808 bp *Sac*II-*Stu*I fragment of pBac/Frag-C^PRVABC-59^, and (v) the 433 bp *Stu*I-*Not*I fragment of pRs/3′RO^PRVABC-59^.

A total of five bacterial expression plasmids were constructed, each of which was used to express a 32 to 51 aa non-hydrophobic region of the ZIKV polyprotein as a glutathione *S*-transferase (GST) fusion protein. In all cases, a defined region of the ZIKV ORF was amplified by PCR using pBac/PRVABC-59 as a template and the appropriate pair of primers listed in Table 2: (i) Frag-zC (147 bp), ZikaC-F + ZikaC-R; (ii) Frag-zM (120 bp), ZikaM-F + ZikaM-R; (iii) Frag-zE (147 bp), ZikaE-F + ZikaE-R; (iv) Frag-zNS4A (177 bp), ZikaNS4A-F + ZikaNS4A-R; and (v) Frag-zNS4B (177 bp), ZikaNS4B-F + ZikaNS4B-R. Each of the resulting amplicons was cloned into pGex-4T-1 (GE Healthcare, Piscataway, NJ, USA) by ligating the 4954 bp *Eco*RI-*Xho*I fragment of the pGex-4T-1 vector with 135, 108, 135, 165, and 165 bp *Eco*RI-*Xho*I fragments of the Frag-zC, -zM, -zE, -zNS4A, and -zNS4B amplicons, respectively. This created pGex-zC, -zM, -zE, -zNS4A, and -zNS4B.

### 2.4. Transcription and Transfection

Infectious transcripts were synthesized from *Psr*I/*Bar*I-linearized BAC plasmid DNA with SP6 RNA polymerase as described [53] in reactions containing m^7^GpppA (New England Biolabs, Ipswich, MA, USA). RNA integrity was examined by agarose gel electrophoresis. RNA was transfected into Vero cells by electroporation using the BTX ECM 830 electroporator with a 2-mm-gap cuvette under optimized conditions (980 V, 99 μs pulse length, and 3 pulses); RNA infectivity was quantified by infectious center assay [55,56]. The infectious centers of plaques/foci formed on the monolayer of Vero cells were visualized at 5 days after transfection either nonspecifically by counterstaining of uninfected cells with crystal violet [55] or specifically by immunostaining of ZIKV-infected cells with rabbit anti-ZIKV NS1 (α-ZNS1) antiserum and horseradish peroxidase-conjugated goat α-rabbit IgG (Jackson ImmunoResearch, West Grove, PA, USA), followed by developing with 3,3′-diaminobenzidine [56].

### 2.5. Growth Kinetics and Cytopathogenicity

Viral growth kinetics and cytopathogenicity were analyzed in 17 animal cell lines from 12 different species. In each case, naïve cells were seeded into 35 mm culture dishes at a density of 3 × 10^5^ cells/dish for 12 h, and then mock-infected or infected with viruses at an MOI of 1 for 1 h at 37 °C. Following incubation, cell monolayers were washed and incubated with complete medium. At 6, 12, 18, 24, 36, 48, 60, 72, and 96 h post-infection (hpi), ZIKV-infected cells were examined morphologically under a light-inverted microscope (Primo Vert, Carl Zeiss, Jena, Germany) to assess the degree of ZIKV-induced cytopathic effect (CPE) as compared to mock-infected cells, and culture supernatants were collected to evaluate the levels of virus production by plaque assays on Vero cells, as described [57]. The infectious centers of plaques were visualized at 5 days after infection by counterstaining of uninfected cells with crystal violet [55].

### 2.6. Real-Time RT-PCR

ZIKV RNA levels in infected Vero cells were quantified as described [57] by real-time RT-PCR with the primer pairs and fluorogenic probes listed in Table 2: the ZikaF + ZikaR and ZikaProbe specific for the ZIKV NS3-coding region that has the identical sequences in all three ZIKVs, and the VeroF + VeroR and VeroProbe specific for the Vero β-actin coding region. Each ZIKV RNA level was normalized to the corresponding β-actin mRNA level as an internal control.

### 2.7. Immunoblotting, Confocal Microscopy, and Flow Cytometry

Individual ZIKV proteins were identified by immunoblotting [31] using each of our six previously characterized JEV region-specific rabbit antisera that cross-react with their ZIKV counterparts, or seven newly generated ZIKV region-specific rabbit antisera. The rabbit antibody was detected using alkaline phosphatase (AP)-conjugated goat α-rabbit IgG (Jackson ImmunoResearch, West Grove, PA, USA), and the AP enzyme was visualized using colorimetric detection with 5-bromo-4-chloro-3-indolyl phosphate and nitro blue tetrazolium (Sigma, St. Louis, MO, USA). ZIKV E proteins were visualized by confocal microscopy [57] with rabbit α-ZE antiserum, followed by secondary labeling with fluorescein isothiocyanate-conjugated goat α-rabbit IgG (Jackson ImmunoResearch). ZIKV NS4A proteins were detected by flow cytometry [58] with rabbit α-ZNS4A antiserum, followed by secondary labeling with Alexa 488-conjugated goat α-rabbit IgG (Invitrogen, Carlsbad, CA, USA).

### 2.8. Mouse Studies

ZIKV neuropathogenicity was examined in male and female mice of four strains: CD-1 (1, 2, and 4 weeks, Charles River, Wilmington, MA, USA), C57BL/6J (4 weeks, the Jackson Laboratory, Bar Harbor, ME, USA), A129 (4 weeks, bred in-house), and AG129 (4 weeks, bred in-house). Groups of mice were inoculated via the intramuscular (im, 50 µL) or intracerebral (ic, 20 µL) route with 10-fold serial dilutions of virus stock in α-minimal essential medium and monitored for any ZIKV-induced clinical signs, weight loss, or death daily for 20 days. The im and ic lethal dose 50% (LD_50_) values for each virus were calculated from the respective dose-dependent survival curves of the infected mice, as described [57].

### 2.9. Ethics Statement

All mouse studies were conducted in strict accordance with the Guide for the Care and Use of Laboratory Animals of the National Institutes of Health, United States of America. The animal protocol was approved by the Institutional Animal Care and Use Committee (IACUC) of Utah State University (approved IACUC protocol #2505, 30/03/2016). Discomfort, distress, pain, and injury were minimized as much as possible through limited handling and euthanization of mice when they were moribund.

## 3. Results

### 3.1. Characterization of Three Spatiotemporally Distinct and Genetically Divergent ZIKV Strains

**Figure 1:**
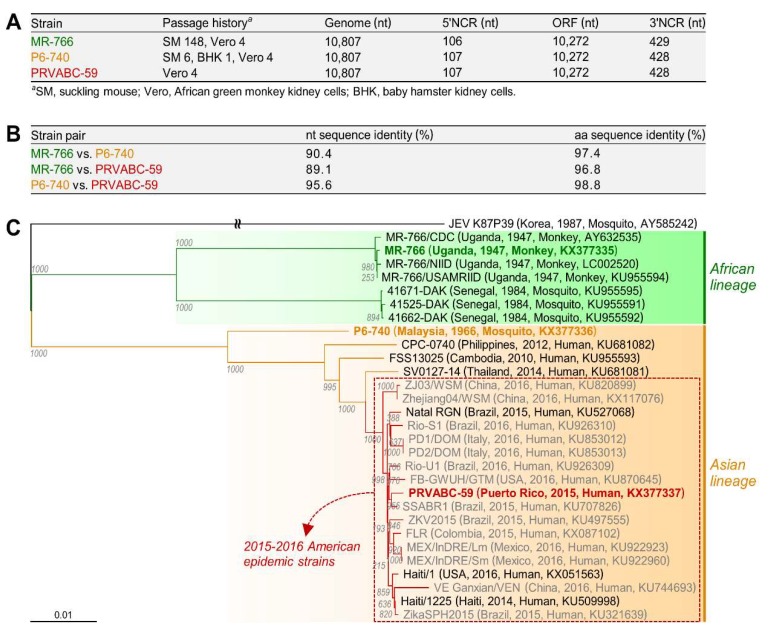
A spectrum of ZIKV genetic diversity is represented by three historically important and spatiotemporally distinct strains: MR-766, P6-740, and PRVABC-59. The consensus nucleotide sequence for each of their full-length genomic RNAs was determined by sequencing three overlapping uncloned cDNA amplicons collectively representing the entire genomic RNA, except for the 5′ and 3′ termini, which were subsequently defined by performing both 5′- and 3′-rapid amplification of cDNA ends (RACE); each of these RACEs was followed by cDNA cloning and sequencing of ~20 randomly picked clones. (**A**) Genomic organization of the three ZIKV strains; (**B**) Pairwise comparison of the complete nucleotide (nt) and deduced amino acid (aa) sequences of the three ZIKV genomes; (**C**) Phylogenetic tree based on the nucleotide sequence of 29 ZIKV genomes, including the 15 complete (MR-766, green; P6-740, orange; PRVABC-59, red; and 12 others, black) and 14 near-complete (gray) genomes, with Japanese encephalitis virus (JEV) K87P39 included as an outgroup. Bootstrap values from 1000 replicates are shown at each node of the tree. The scale bar represents the number of nucleotide substitutions per site. The strain name is followed by a description in parenthesis of the country, year, and host of isolation and the GenBank accession numbers. Note that MR-766 has been fully sequenced in this study and by three other groups (designated MR-766/CDC, MR-766/NIID, and MR-766/USAMRIID).

As an initial step in examining the genetic diversity of ZIKV and its biological significance for viral replication and pathogenesis, we selected three historically important strains of distinct geographical and temporal origins: (i) MR-766, the first ZIKV identified from the blood of a rhesus macaque monkey in Uganda in 1947 [1]; (ii) P6-740, the first non-African strain, isolated from a pool of *A. aegypti* mosquitoes in Malaysia in 1966 [59]; and (iii) PRVABC-59, the recent American strain recovered from the blood of a human patient in Puerto Rico in 2015 [60]. To compare the genome sequence and composition of these three ZIKVs, we determined the consensus nucleotide sequence for each of their full-length genomic RNAs [54]. In all three strains of ZIKV, we found that the genomic RNA is 10,807 nt long, with a single ORF of 10,272 nt flanked by a 106 or 107 nt 5′NCR and a 428 or 429 nt 3′NCR (Figure 1A). Also, the three genomic RNAs all begin with the dinucleotide 5′-AG and end with the dinucleotide CU-3′, both of which are conserved among all mosquito- and tick-borne flaviviruses. However, pairwise sequence comparisons of the three complete genomes showed a considerable degree of genetic diversity, with a range in sequence identity of 89.1–95.6% at the nucleotide level and 96.8–98.8% at the amino acid level over the 3423 aa polyprotein encoded by the single ORF of the genomic RNA (Figure 1B).

To examine the genetic relationship between the three spatiotemporally distinct ZIKVs and their associations with other strains, we performed a multiple sequence alignment for phylogenetic analysis using the nucleotide sequence of all 29 ZIKV genomes (15 complete, 14 near-complete) in GenBank at the time of analysis (June 2016), including our complete nucleotide sequence of the genomes of MR-766, P6-740, and PRVABC-59. Construction of a genome-based rooted phylogenetic tree using JEV K87P39 as an outgroup revealed two distinct phylogenetic groups (Figure 1C), in agreement with previous ORF-based phylogenetic studies that classified 10–40 ZIKV isolates into two major genetic lineages, African and Asian [40,41,42]. The African lineage branches into two clusters, one including four different versions of the Ugandan MR-766 strain (1947) that are not identical in genome sequence, mainly because of a variation in the passage history of the virus, and the other including the three Senegalese isolates 41671-DAK, 41525-DAK, and 41662-DAK, all isolated in 1984. On the other hand, the Asian lineage contains a single cluster of the Malaysian P6-740 (1966), Cambodian FSS13025 (2010), Philippine CPC-0740 (2012), and Thai SV0127-14 (2014) strains, as well as 18 other isolates collected during the 2015–2016 American epidemic, including the Puerto Rican PRVABC-59 strain (2015). Notably, the four pre-epidemic Asian strains (P6-740, FSS13025, CPC-0740, and SV0127-14) are closely related to the 2015–2016 American epidemic strains, but each forms a single minor branch. Overall, our data indicate that MR-766 belongs to the African lineage, whereas both P6-740 and PRVABC-59 belong to the Asian lineage, with PRVABC-59 being derived from an ancestor of the Asian lineage.

### 3.2. Development of Genetically Stable Full-Length Infectious cDNA Clones for the Three ZIKV Strains

**Figure 2:**
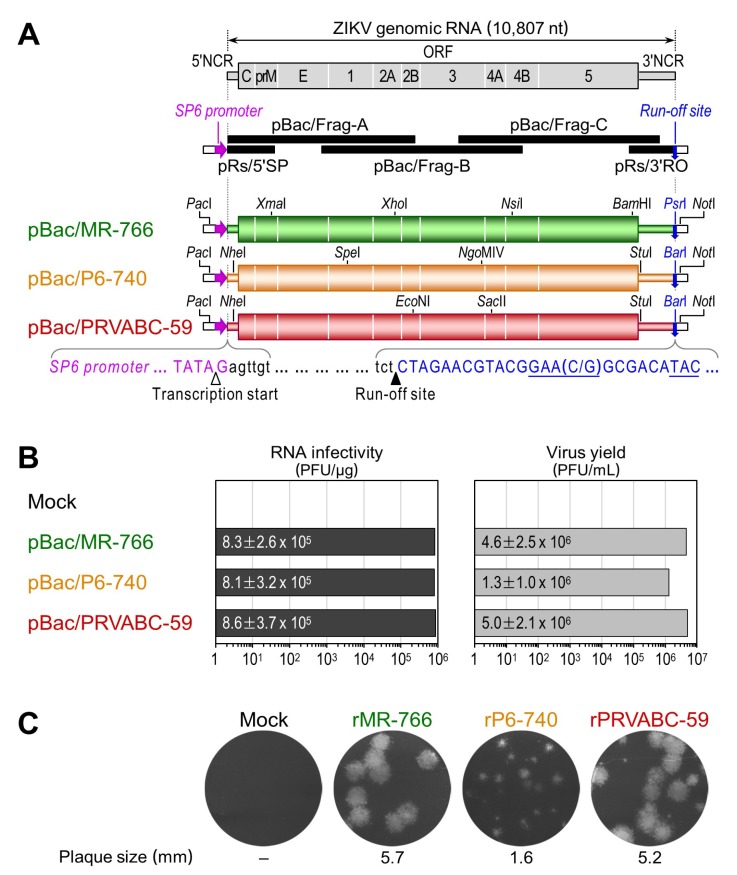
A trio of functional ZIKV cDNAs was created for the rescue of three molecularly cloned genetically divergent strains: rMR-766, rP6-740, and rPRVABC-59. (**A**) Construction of three full-length ZIKV cDNAs as BACs for MR-766, P6-740, and PRVABC-59. In all three cases, each genomic RNA (top panel) was first subcloned into five overlapping cDNAs (middle panel), which were then joined at four shared restriction sites as indicated to assemble its full-length cDNA without introducing any point mutations for cloning (bottom panel). Presented below the three full-length cDNAs are the sequences corresponding to the 5′ and 3′ termini conserved in all three ZIKVs (black lowercase), an SP6 promoter placed just upstream of the viral genome (magenta uppercase), and a run-off site positioned immediately downstream of the viral genome (*Psr*I or *Bar*I, blue uppercase). Marked below the sequences are the transcription start (white arrowhead) and run-off (black arrowhead) sites; (**B**) Functionality of the three full-length ZIKV cDNAs. After linearization with *Psr*I or *Bar*I, as appropriate, each full-length cDNA was used as a template for in vitro transcription with SP6 RNA polymerase in the presence of the dinucleotide cap analog m^7^GpppA. Capped RNA transcripts were transfected into Vero cells to determine the number of infectious centers (plaques) counterstained with crystal violet at 5 days after transfection (RNA infectivity). At 36 h post-transfection, culture supernatants from RNA-transfected cells were harvested to estimate the level of virus production by plaque assays on Vero cells (Virus yield). Means and standard deviations from three independent experiments are shown; (**C**) Plaque morphology. The average plaque sizes were estimated by measuring 20 representative plaques.

We constructed three full-length infectious ZIKV cDNAs for the MR-766, P6-740, and PRVABC-59 strains, each capable of serving as a template for the rescue of molecularly cloned ZIKVs (Figure 2). In each strain, five overlapping cDNA fragments representing the 10,807 nt genomic RNA were sequentially assembled into a full-length cDNA in the single-copy BAC vector pBeloBAC11, in order to ensure the stable maintenance of the cloned cDNA during propagation in *Escherichia coli*, with an SP6 promoter sequence positioned immediately upstream of the viral 5′-end and a unique restriction endonuclease recognition site (*Psr*I for MR-766, *Bar*I for P6-740 and PRVABC-59) placed just downstream of the viral 3′-end. Both the SP6 promoter and the unique restriction site were engineered so that in vitro run-off transcription could be used to produce m^7^G-capped synthetic RNAs bearing authentic 5′ and 3′ ends of the viral genomic RNA. Using this BAC-based cloning strategy, we thus created a panel of three full-length ZIKV cDNAs, designated pBac/MR-766, pBac/P6-740, and pBac/PRVABC-59 (Figure 2A).

To evaluate the functionality of the three full-length ZIKV BACs, we determined the viability of the synthetic RNAs transcribed in vitro from each BAC by measuring their specific infectivity after RNA transfection into a ZIKV-susceptible African green monkey kidney (Vero) cell line. To prepare a DNA template for in vitro run-off transcription, the three full-length ZIKV BACs were first linearized by digestion with *Psr*I (for pBac/MR-766) or *Bar*I (for pBac/P6-740 and pBac/PRVABC-59). Each was then used as a template for a run-off transcription reaction using SP6 RNA polymerase in the presence of the m^7^GpppA cap structure analog. After removal of the DNA template by DNase I digestion, we transfected Vero cells with the RNA transcripts, quantifying their infectivity as the number of plaque-forming units (PFU) per μg of transfected RNA. In all three BACs, the RNA transcripts invariably had a high infectivity of 8.1–8.6 × 10^5^ PFU/μg and were capable of producing a high-titer stock of infectious ZIKVs in culture medium that reached 1.3–5.0 × 10^6^ PFU/mL at 36 h after transfection (Figure 2B). Each of the three recombinant BAC-derived ZIKVs (designated by the prefix “r”) formed a homogeneous population of plaques that differed from the others in size, with mean diameters of 5.7 (rMR-766), 1.6 (rP6-740), and 5.2 (rPRVABC-59) mm (Figure 2C). We also demonstrated that using pBac/P6-740, the infectivity of its RNA transcripts was decreased by ~4 logs to a barely detectable level (55–105 PFU/μg), with a single C^9804^→U substitution (an unintended mutation introduced during the overlapping cDNA synthesis by RT-PCR) replacing a His with Tyr at position 713 of the viral NS5 protein (Figure S1A,B). On the crystal structure of ZIKV NS5 [61,62], the His-713 residue is located within the conserved structural motif E region near the priming loop in the RNA-dependent RNA polymerase domain (Figure S1C), suggesting a critical role for His-713 in the polymerase function of ZIKV NS5.

Next, we examined the genetic stability of the three full-length ZIKV BACs that are important for reliable and efficient recovery of infectious viruses from the cloned cDNAs. A single colony of *E. coli* DH10B carrying each of the three full-length ZIKV BACs was grown in liquid medium and serially passaged for 4 days by diluting it 10^6^-fold daily, such that each passage represented ~20 generations, as we described previously [53]. In all three cases, we found no differences in specific infectivity of the RNA transcripts made from the BAC plasmids even after 80 generations, indicating that the three full-length ZIKV BACs are stable during propagation in bacteria (Figure S2). In sum, we have established genetically stable BAC-based reverse genetics platforms for the recovery of three molecularly cloned, genetically distinct ZIKVs.

### 3.3. Differential Replication Kinetics and Cytopathogenicity among Three Molecularly Cloned ZIKVs in Human, Mosquito, and Animal Cell Lines

**Figure 3:**
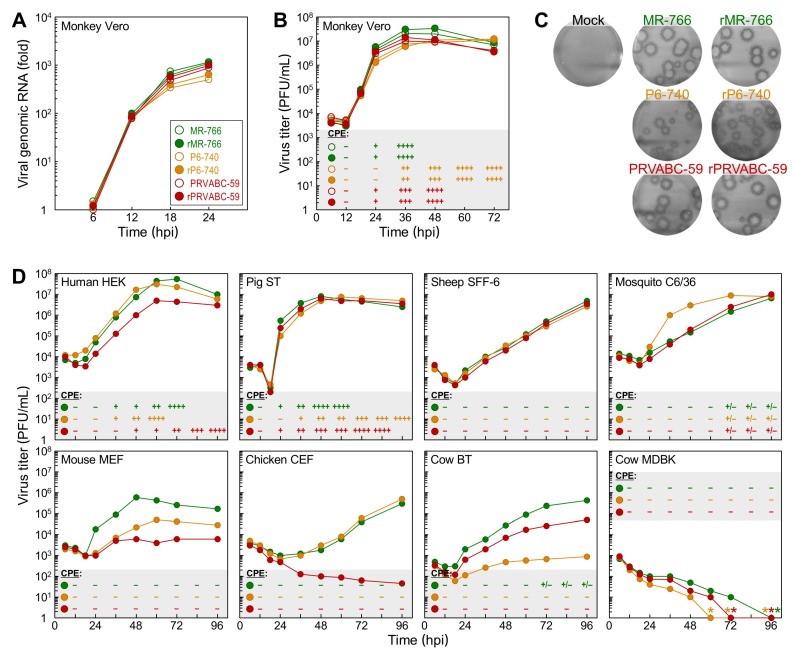
ZIKV replication kinetics and cytopathogenicity in cell cultures depend on the particular combination of virus strain and host cells. (**A**–**C**) Replicative and cytopathic properties of three cloned cDNA-derived ZIKVs (rMR-766, rP6-740, and rPRVABC-59) and their uncloned parental ZIKVs (MR-766, P6-740, and PRVABC-59) in Vero cells. Cells were infected with each of the six ZIKVs (MOI = 1). At the time points indicated after infection, cells were lysed to examine the accumulation levels of viral genomic RNA by real-time RT-PCR with a ZIKV-specific fluorogenic probe (**A**), and supernatants were collected to analyze the production levels of progeny virions by plaque assays on Vero cells (**B**). At 5 days post-infection, cell monolayers maintained under a semisolid overlay medium were immunostained with rabbit α-ZNS1 antiserum to visualize the infectious foci (**C**). (**D**) Growth kinetics and cytopathogenicity of the three cloned cDNA-derived ZIKVs in a wide range of animal cells (see also Figure S3). Each virus was used to infect the cell lines (MOI = 1) specified in the figure. At the indicated time points, cells were examined microscopically for the degree of ZIKV-induced cytopathic effect (CPE) (–, 0%; +, 0–25%; ++, 25–50%; +++, 50–75%; ++++, 75–100% cell death), and supernatants were assayed for virus production by plaque assays on Vero cells. hpi, hours post-infection.

To test whether the genetic variation in ZIKV can have differential effects on its replication kinetics and cytopathogenicity, we infected monkey kidney-derived Vero cells at an MOI of 1, then examined the replicative and cytopathic properties of the three cloned cDNA-derived ZIKVs (rMR-766, rP6-740, and rPRVABC-59) as compared to those of the uncloned parental ZIKVs (MR-766, P6-740, and PRVABC-59) used for cDNA construction. In all three strains, we saw no noticeable differences between the cloned and uncloned viruses in the accumulation of viral genomic RNA over the first 24 hpi (Figure 3A), paralleling not only the kinetics of viral growth and CPE development over the first 3 days post-infection (Figure 3B) but also the average sizes of the α-ZNS1 antibody-reactive foci immunostained at 5 days post-infection (Figure 3C). However, we did observe clear differences between the three strains, regardless of whether they were cloned or uncloned viruses, with respect to their replication kinetics and cytopathogenicity (Figure 3A–C). Our specific findings are as follows: (i) rMR-766/MR-766 displayed the fastest rate of RNA replication, induced complete lysis of the infected cells by 36 hpi, achieved the highest virus titer of 2.0–3.3 × 10^7^ PFU/mL at 36–48 hpi, and formed the largest foci of 6.3 mm diameter. (ii) rP6-740/P6-740 had the slowest rate of RNA replication, did not cause complete CPE until 72 hpi, reached its maximal virus titer of 1.1–1.2 × 10^7^ PFU/mL at 60–72 hpi, and generated the smallest foci of 2.4 mm diameter. (iii) rPRVABC-59/PRVABC-59 had a rate of RNA replication slightly slower than rMR-766/MR-766 but much faster than that of rP6-740/P6-740; it caused complete CPE by 48 hpi, with a peak virus titer of 0.9–1.4 × 10^7^ PFU/mL at 36–48 hpi, and produced foci of 5.9 mm diameter.

We further analyzed the replicative and cytopathic potential of the three cDNA-derived ZIKVs in 16 other animal cell lines from 11 different species, over the first 4 days after infection of the cells with each virus at an MOI of 1. Our data revealed seven distinct patterns of viral growth kinetics and cytopathogenesis, depending on a combination of the viral strain and host cell line (Figure 3D and Figure S3): (1) In all three human cell types (embryonic kidney HEK, hepatocarcinoma Huh-7, and neuroblastoma SH-SY5Y), rMR-766 and rP6-740 grew equally well, to maximum titers of 10^7^–10^8^ PFU/mL at 48–72 hpi, but rPRVABC-59 always grew at a slower rate, attaining a peak titer 1–2 logs lower than that of the other two strains at 72–96 hpi (HEK and SH-SY5Y) or reaching a peak titer similar to that of the other two strains only at 96 hpi (Huh-7); all three ZIKVs induced cell death, with a correlation between the degree of CPE and the magnitude of viral replication. (2) In swine testis (ST) and equine skin (NBL-6) cells, the three ZIKVs replicated to their peak titers of 10^6^–10^7^ PFU/mL at 48 hpi, with differential growth rates similar to those seen in Vero cells (rMR-766, fastest; rP6-740, slowest; rPRVABC-59, intermediate) that paralleled the kinetics of CPE development. (3) In sheep fetal fibroblast (SFF-6) and *A. albopictus* (C6/36) cells, the three ZIKVs shared a superimposable growth curve, characterized by a steady increase in virus titers up to ~10^7^ PFU/mL by 96 hpi, except for rP6-740, which had an exponential growth during 24–48 hpi in C6/36, but not SFF-6 cells. None of the three ZIKVs produced any visible CPE. (4) In goat fetal fibroblast (GFF-4), canine kidney (MDCK), and feline kidney (CRFK) cells and in all three mouse cell types (C57BL/6-derived embryonic fibroblast MEF, NIH/Swiss-derived embryonic fibroblast NIH/3T3, and motor neuron-like hybrid NSC-34), rMR-766 was the fastest-growing, reaching its highest titer of 10^6^–10^7^ PFU/mL at 48–96 hpi; rPRVABC-59 was the slowest-growing, gaining a maximum titer of only 10^3^–10^4^ PFU/mL during the same period; and rP6-740 was intermediate in growth rate. However, none of these viruses produced visible CPE. (5) In chicken embryo fibroblast (CEF) cells, both rMR-766 and rP6-740 had a relatively long lag period of 36 h, followed by a gradual increase in virus titer up to 10^5^–10^6^ PFU/mL by 96 hpi; in contrast, rPRVABC-59 grew extremely poorly, resulting in a slow decrease in virus titer to 45 PFU/mL by 96 hpi. No CPE was observed for any of the three ZIKV-infected CEF cells. (6) In bovine turbinate (BT) cells, the three ZIKVs showed substantial differences in growth kinetics, reaching a plateau at 96 hpi, with peak titers of 4.4 × 10^5^ (rMR-766), 5.0 × 10^4^ (rPRVABC-59), and 8.8 × 10^2^ (rP6-740) PFU/mL. However, no visible CPE was induced in any of the ZIKV-infected cells. (7) In bovine kidney (MDBK) cells, the titers of all three ZIKVs declined to undetectable levels at 60–96 hpi, with no overt signs of viral replication.

**Figure 4:**
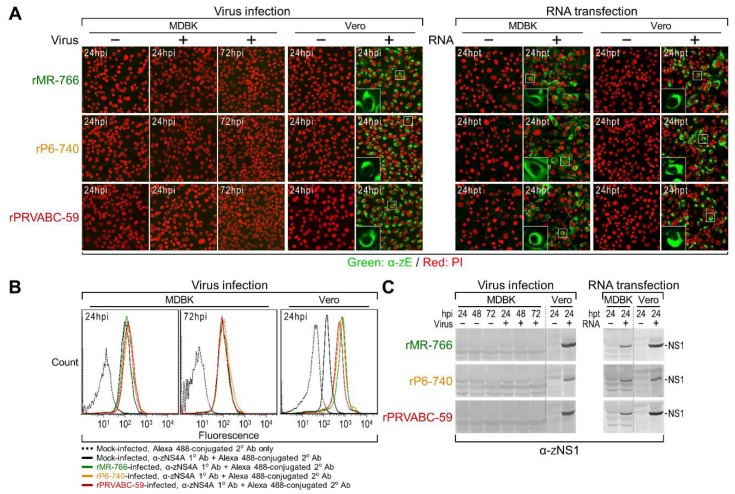
MDBK cells are permissive for ZIKV RNA replication but are not susceptible to infection with the virus. MDBK cells were mock-infected or infected with rMR-766, rP6-740, or rPRVABC-59 at an MOI of 3 (for virus infection experiments), or mock-transfected or transfected with 3 µg of synthetic RNAs transcribed in vitro from their respective infectious cDNAs (for RNA transfection experiments). At the indicated time points, the expression of three ZIKV proteins (E, NS1, and NS4A) within the cells was analyzed by confocal microscopy for E (**A**), flow cytometry for NS4A (**B**), and immunoblotting for NS1 (**C**). The insets in panel A show enlarged views of the boxed areas with the fluorescence of propidium iodide (PI)-stained nuclei excluded. In all experiments, ZIKV-susceptible Vero cells were included in parallel. hpi, hours post-infection; hpt, hours post-transfection.

Subsequently, we showed that MDBK cells are not susceptible to ZIKV infection, but instead are permissive for ZIKV RNA replication, by using (i) single cell-based immunofluorescence (Figure 4A) and flow cytometry (Figure 4B) assays to determine the number of cells expressing ZIKV proteins (E or NS4A), when MDBK cells were either infected with each of the three cDNA-derived ZIKVs or transfected with each of the three infectious RNAs transcribed in vitro from their corresponding cDNAs; and (ii) total cell lysate-based immunoblot analyses to assess the accumulation levels of ZIKV NS1 protein in the virus-infected vs RNA-transfected MDBK cells (Figure 4C). In all these experiments, we used Vero cells, a ZIKV-susceptible cell line, as a control. Our results led us to postulate that MDBK cells might lack one or more host factors required for ZIKV entry; alternatively, they might have a general defect in the clathrin-dependent endocytic pathway that ZIKV utilizes for internalization [63]. We thus investigated the functional integrity of the clathrin-dependent endocytic pathway in MDBK cells, by analyzing the susceptibility of these cells to infection by two other enveloped RNA viruses whose entry depends on clathrin-mediated endocytosis: bovine viral diarrhea virus (BVDV) and vesicular stomatitis virus (VSV). In contrast to their resistance to ZIKV infection, we found that MDBK cells were highly susceptible to infection with both BVDV and VSV, as demonstrated by their plaque formation and high level of progeny virion production (Figure S4A,B). These results indicate that the cellular machinery associated with the clathrin-dependent endocytic pathway is functional in MDBK cells, and they support our hypothesis that MDBK cells lack a host factor(s) promoting ZIKV entry.

### 3.4. Genome-Wide Landscape of the Viral Gene Products and Their Related Species Produced by the Molecularly Cloned ZIKVs

**Figure 5:**
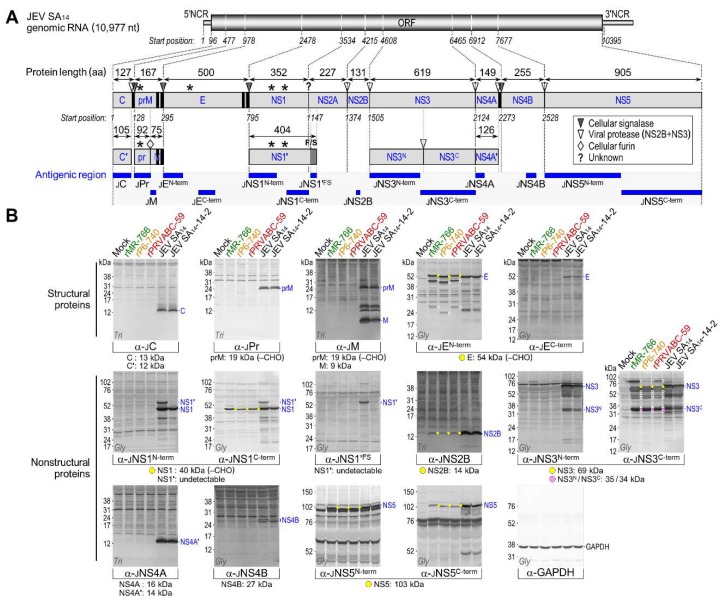
A subset of 15 JEV region-specific polyclonal antibodies detects the cross-reactive ZIKV E, NS1, NS2B, NS3, NS5, and their related species in ZIKV-infected cells. (**A**) Schematic illustration showing the antigenic regions recognized by 15 JEV region-specific rabbit antisera. The 10,977 nt genomic RNA of JEV SA_14_ has a 95 nt 5′NCR, a 10,299 nt ORF, and a 583 nt 3′NCR (top panel). The ORF encodes a 3432 aa polyprotein that is processed by viral and cellular proteases into at least 10 mature proteins (middle panel). Marked on the polyprotein are one or two transmembrane domains (vertical black bar) at the C-termini of three structural proteins (C, prM, and E) and at the junction of NS4A/NS4B, as well as four *N*-glycosylation sites (asterisk) in the pr portion of prM (^15^NNT), E (^154^NYS), and NS1 (^130^NST and ^207^NDT). During viral morphogenesis, prM is cleaved by furin protease into a soluble pr peptide and a virion-associated M protein. NS1′ is the product of a −1 ribosomal frameshift (F/S) event that occurs at codons 8–9 of NS2A, adding a 52 aa C-terminal extension to the NS1 protein. The bottom panel displays the antigenic regions (horizontal blue bar) recognized by 15 JEV region-specific rabbit antisera. (**B**) Identification of viral proteins in ZIKV-infected cells by immunoblotting. Vero cells were mock-infected or infected at MOI 1 with each of three ZIKVs (rMR-766, rP6-740, and rPRVABC-59) or two JEVs (SA_14_ and SA_14_-14-2, for reference). At 20 h post-infection, total cell lysates were separated by SDS-PAGE on a glycine (Gly) or tricine (Tri) gel and analyzed by immunoblotting with each of the 15 JEV region-specific rabbit antisera or α-GAPDH rabbit antiserum as a loading and transfer control. Molecular size markers are given on the left of each blot, and major JEV proteins for reference are labeled on the right. Provided below each blot are the estimated molecular sizes of the predicted ZIKV proteins, and marked on the blot are the predicted proteins (yellow or pink dot) and presumed cleavage intermediates or further cleavage/degradation products (white circle). CHO, *N*-glycosylation.

**Figure 6:**
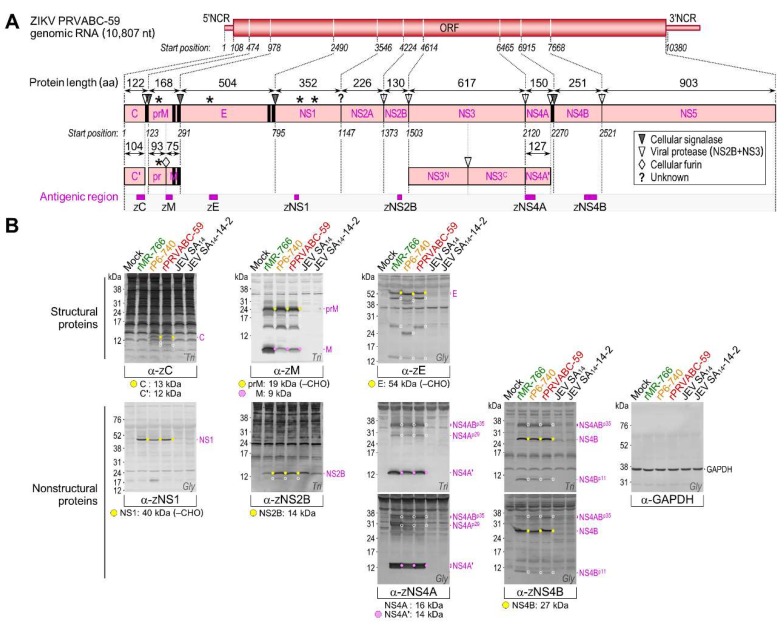
A panel of seven ZIKV region-specific polyclonal antibodies identifies ZIKV C, prM/M, E, NS1, NS2B, NS4A’, NS4B, and their related species in ZIKV-infected cells. (**A**) Schematic illustration showing the antigenic regions recognized by seven ZIKV region-specific rabbit antisera. The 10,807 nt genomic RNA of ZIKV PRVABC-59 consists of a 107 nt 5′NCR, a 10,272 nt ORF, and a 428 nt 3′NCR (top panel). The ORF encodes a 3423 aa polyprotein that is predicted to be cleaved by viral and cellular proteases into at least 10 mature proteins (middle panel). Marked on the polyprotein and its products are one or two transmembrane domains (vertical black bar) at the C-termini of three structural proteins (C, prM, and E) and at the junction of NS4A/NS4B, as well as four *N*-glycosylation sites (asterisk) in the pr portion of prM (^70^NTT), E (^154^NDT), and NS1 (^130^NNS and ^207^NDT). The bottom panel shows the antigenic regions (horizontal magenta bar) recognized by seven ZIKV region-specific rabbit antisera. (**B**) Identification of viral proteins in ZIKV-infected cells by immunoblotting. Vero cells were mock-infected or infected at MOI 1 with each of three ZIKVs (rMR-766, rP6-740, and rPRVABC-59) or two JEVs (SA_14_ and SA_14_-14-2, for comparison). At 20 h post-infection, total cell lysates were separated by SDS-PAGE on a glycine (Gly) or tricine (Tri) gel and analyzed by immunoblotting with each of the seven ZIKV region-specific rabbit antisera or α-GAPDH rabbit antiserum as a loading and transfer control. Molecular size markers are given on the left of each blot, and major ZIKV proteins are labeled on the right. Provided below each blot are the estimated molecular sizes of predicted ZIKV proteins, and marked on the blot are the predicted proteins (yellow or pink dot) and presumed cleavage intermediates or further cleavage/degradation products (white circle). CHO, *N*-glycosylation.

To identify all the viral proteins produced by rMR-766, rP6-740, and rPRVABC-59, we examined total cell lysates of mock- and ZIKV-infected Vero cells in two series of immunoblotting experiments. In the first series, we probed with each of our 15 JEV region-specific rabbit antisera (Figure S5), originally produced to detect all JEV gene products [31], which we estimated to have the potential for cross-reactivity with their ZIKV counterparts, given the relatively high levels (35–71%) of amino acid sequence identity between their antigenic regions (Figure 5A). Indeed, six (α-JE^N-term^, α-JNS1^C-term^, α-JNS2B, α-JNS3^C-term^, α-JNS5^N-term^, and α-JNS5^C-term^) of the 15 antisera showed moderate-to-strong cross-reactivity with their respective ZIKV gene products, but the remaining nine had no reactivity (Figure 5B). To cover the remaining undetected parts of ZIKV ORF, we then generated seven ZIKV region-specific rabbit antisera, using rPRVABC-59 as the viral strain of choice, immunizing the rabbits with five bacterially expressed GST fusion proteins (α-ZC, α-ZM, α-ZE, α-ZNS4A, and α-ZNS4B) or two chemically synthesized oligopeptides (α-ZNS1 and α-ZNS2B) (Figure S6A,B). In all cases, the 19 to 51 aa antigenic regions of ZIKV were selected to have relatively low levels (16–42%) of amino acid sequence identity with those of JEV (Figure 6A). The resulting seven ZIKV region-specific antisera were used for a second series of immunoblots, in which we detected their respective ZIKV gene products (Figure 6B). In all immunoblots, we included two additional cell lysates (as a reference for JEV proteins) extracted from Vero cells infected with the virulent JEV strain SA_14_ or its attenuated strain SA_14_-14-2; both JEVs share the same genome-wide viral protein expression profile, except that the NS1′ protein is expressed only by SA_14_ [57].

Our immunoblot analysis using a collection of 13 ZIKV antigen-reactive region-specific rabbit antisera allowed us to create a full catalog of viral gene products and their related species, except for the predicted 24 kDa NS2A (Figure 5 and Figure 6): (1) α-ZC recognized the 13 kDa C protein, with no accumulation of the further-processed 12 kDa C’ (see below for description of virion-associated proteins), but with appearance of one or two cleavage products of 10–11 kDa in rPRVABC-59- or rP6-740-infected cells, respectively; however, this antiserum did not react with any of the C-related proteins of rMR-766. (2) α-ZM reacted strongly with the 9 kDa M protein and its 24 kDa precursor prM, with the ratio of M:prM varying in the presence of equal amounts of the loading control GAPDH protein, depending on the viral strain; the observed size of prM was 5 kDa larger than its predicted size, consistent with an addition of *N*-glycans at Asn-70 (^70^NTT) to its pr domain [64] that is conserved in all three ZIKVs. Also, the α-ZM reacted weakly with at least two minor proteins of 15 and 19 kDa. (3) α-JE^N-term^/α-ZE detected four E-related proteins (of 54/56, 43/45, 24/26, and 14 kDa). Among these, the first three proteins from rP6-740 were all 2 kDa smaller than those from rMR-766 and rPRVABC-59, in agreement with a missense mutation of the *N*-glycosylation site at Asn-154 (^154^NDT→NDI) in the E protein of rP6-740 relative to that of rMR-766 and rPRVABC-59 [19,20]. Indeed, the three 2 kDa smaller proteins from rP6-740 became similar in size to those from rMR-766 and rPRVABC-59, when the mutated *N*-glycosylation motif in rP6-740 was restored by changing ^154^NDI into ^154^NDT, but not by changing ^154^NDI into ^154^QDT (Figure S7A–C). (4) Both α-JNS1^C-term^ and α-ZNS1 identified the 45 kDa NS1 exclusively. This protein was 5 kDa larger than predicted by its amino acid sequence because of the addition of *N*-glycans at Asn-130 (^130^NNS) and Asn-207 (^207^NDT), both of which are conserved in all three ZIKVs [65,66]. As expected, these data also showed that only NS1, and not its frameshift product NS1’, was produced by all three ZIKVs. (5) α-JNS2B/α-ZNS2B revealed the 14 kDa NS2B, together with a minor protein of 11 kDa at a barely detectable level. (6) α-JNS3^C-term^ recognized the 69 kDa NS3; it also reacted more strongly with a major cleavage product of 34 kDa, representing the C-terminal half of the full-length NS3 [31], and less intensely with at least seven minor proteins of 33–60 kDa. Intriguingly, α-JNS3^C-term^ detected a species with a mass of 85 kDa, corresponding to the calculated size of an NS2B-3 or NS3-4A/4A’ processing intermediate. (7) α-ZNS4A did not detect the predicted 16 kDa NS4A, but did predominantly recognize its further-processed 14 kDa NS4A’, which ran as a single species in tricine–SDS-PAGE but migrated as a doublet in glycine–SDS-PAGE. Unexpectedly, this antiserum also identified two clusters of multiple protein bands, one at 29 kDa (NS4A^p29^) and the other at 35 kDa (NS4AB^p35^, which also reacted with α-ZNS4B; Figure S8A,B). (8) α-ZNS4B stained the predicted major 27 kDa NS4B, along with two minor proteins at 11 kDa (NS4B^p11^) and 35 kDa (NS4AB^p35^, which again reacted with α-ZNS4A; Figures S8A,B). (9) α-JNS5^N-term^ and α-JNS5^C-term^ reacted with the predicted 103 kDa NS5.

**Figure 7:**
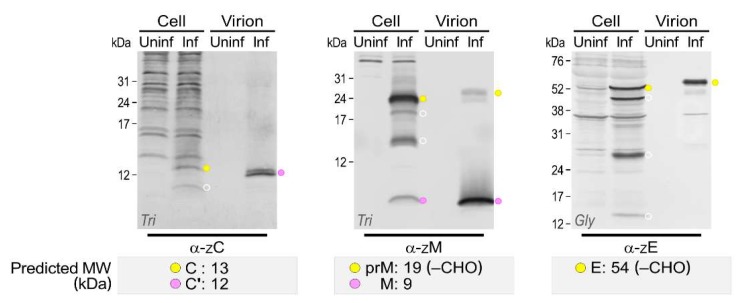
Profiling of virion-associated ZIKV proteins compared to their cell-associated counterparts. Vero cells were left uninfected (Uninf) or infected (Inf) with ZIKV rPRVABC-59 at an MOI of 1. For cell-associated viral proteins, total cell lysates were prepared by lysing the cell monolayers at 20 h post-infection. For virion-associated viral proteins, cell culture supernatants were collected at the same time point, and extracellular virions were pelleted by ultracentrifugation through a 20% sucrose cushion. Equivalent portions of total cell lysates and pelleted virions were resolved by SDS-PAGE on a glycine (Gly) or tricine (Tri) gel and analyzed by immunoblotting with α-ZC, α-ZM, or α-ZE. Molecular weight markers are shown on the left of each blot. The molecular weights of predicted C, C’, prM, M, and E proteins are indicated below each blot. Marked on each blot are the predicted proteins (yellow or pink dot) and presumed further cleavage/degradation products (white circle). CHO, *N*-glycosylation.

In addition to the three full-length structural proteins (C, prM/M, and E) of ZIKV, their multiple smaller products were accumulated to lower but still significant amounts in Vero cells infected with each of the three ZIKVs, with nearly the same protein expression profile (Figure 6). To define the actual viral structural proteins incorporated into ZIKV particles, rPRVABC-59 was used to profile all the structural proteins associated with extracellular virions, which were purified by pelleting through a 20% sucrose cushion. We then compared them with their cell-associated counterparts by immunoblotting with α-ZC, α-ZM, and α-ZE (Figure 7). The purified ZIKV particles were shown to contain (i) the 12 kDa C’ protein, which appeared as a closely spaced doublet with the lower band being more prominent than the upper band and migrating in a gel marginally faster than one cell-associated major 13 kDa C protein, but slower than the other cell-associated minor 10 kDa C-derived cleavage product; (ii) the 9 kDa M protein and a trace amount of its glycosylated precursor prM, which appeared as two bands, the slightly less intense and faster one migrating with a mass of 23–24 kDa and the slightly more intense and slower one at 25–26 kDa, reflecting the trimming of high mannose and the addition of more complex sugars to the cell-associated 24 kDa prM protein during virus release through the cellular secretory pathway [67]; and (iii) the glycosylated 58 kDa E protein, which ran slightly slower than the cell-associated 56 kDa E protein, again reflecting the difference in its glycosylation status. Collectively, we have demonstrated that the extracellular ZIKVs are composed of three post-translationally modified full-length structural proteins, excluding their smaller species.

### 3.5. Wide Range of Differences in Age-Dependent Neuropathogenicity among Three Molecularly Cloned ZIKVs in Outbred CD-1 Mice

We compared the virulence of rMR-766, rP6-740, and rPRVABC-59 in CD-1 mice at three different ages (1, 2, and 4 weeks) by examining two neuropathogenic properties: (i) neuroinvasiveness (the ability to penetrate the central nervous system from a peripheral site), quantified by generating the dose-dependent survival curve and determining the LD_50_ after an im inoculation; and (ii) neurovirulence (the ability to establish a lethal infection within the central nervous system), quantified by creating the dose-dependent survival curve and measuring the LD_50_ after an ic inoculation. For both im and ic inoculations, we first determined the appropriate dose ranges for calculating the LD_50_ values, and we optimized the study designs prior to the performance of full-scale experiments. For these pilot experiments, we injected all three age groups of the mice with a maximum dose of each virus: 1.2 × 10^5^ PFU/mouse for im inoculations and 3.6 × 10^4^ PFU/mouse for ic inoculations. If necessary, we then performed a series of large-scale dose-response studies, inoculating groups of the mice at 1, 2, and 4 weeks of age via the im or ic route with serial 10-fold dilutions of the virus. Following infection, the mice were monitored daily for mortality, weight loss, and other clinical signs of illness over 20 days.

**Figure 8:**
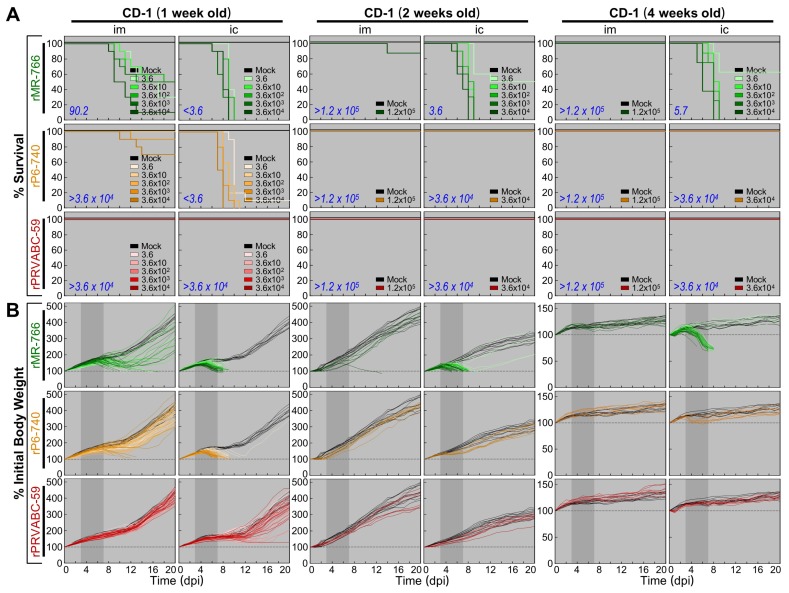
Three molecularly cloned ZIKVs display a full range of variation in neuropathogenicity for outbred CD-1 mice in an age-dependent manner. Groups of CD-1 mice (*n* = 8–10, half male, half female) were mock-inoculated or inoculated at 1, 2, and 4 weeks of age via the intramuscular (im) or intracerebral (ic) route with a maximum dose of 3.6 × 10^4^ or 1.2 × 10^5^ PFU, or serial 10-fold dilutions of rMR-766, rP6-740, or rPRVABC-59. (**A**) Survival curves were generated by the Kaplan–Meier method, and LD_50_ values were determined by the Reed–Muench method and are presented in the bottom left corner of each curve. (**B**) Weight changes are plotted, with each mouse represented by one color-coded line. dpi, days post-infection.

The comparative assessments of our dose-dependent survival curves and LD_50_ values revealed the following (Figure 8A): (i) rMR-766 exhibited age-dependent neuroinvasiveness, as evidenced by an im LD_50_ of 90.2 PFU for 1-week-old mice and >1.2 × 10^5^ PFU for 2- and 4-week-old mice, yet it displayed a high level of neurovirulence at all three ages, as evidenced by an ic LD_50_ of <3.6, 3.6, and 5.7 PFU for 1-, 2-, and 4-week-old mice, respectively. (ii) rP6-740 showed barely detectable neuroinvasiveness in 1-week-old mice, with only 1 or 3 of 10 infected mice dying when inoculated with the two highest doses, 3.6 × 10^3^ or 3.6 × 10^4^ PFU/mouse, respectively (im LD_50_, >3.6 × 10^4^ PFU). Similarly, it had no detectable neuroinvasiveness in 2- and 4-week-old mice, with no infected mice dying even when inoculated with the highest dose, 1.2 × 10^5^ PFU/mouse (im LD_50_, >1.2 × 10^5^ PFU). However, rP6-740 showed age-dependent neurovirulence, as it was highly neurovirulent in 1-week-old mice (ic LD_50_, <3.6 PFU) but non-neurovirulent in 2- and 4-week-old mice (ic LD_50_, >3.6 × 10^4^ PFU). (iii) rPRVABC-59 was essentially non-neuroinvasive and non-neurovirulent, regardless of the mouse age, with its im and ic LD_50_ values estimated to be greater than the highest dose used for each route of infection, without a single death. Of the three ZIKVs, therefore, rMR-766 was the most virulent, rPRVABC-59 was the least virulent, and rP6-740 showed intermediate virulence. The observed differences in virulence are correlated with the variations in viral passage history in mice (Figure 1A).

Moreover, we recognized not only the lethal virulence displayed by rMR-766 and rP6-740 but also the non-lethal virulence exhibited by all of the three ZIKVs, including rPRVABC-59. This effect was most prominent in 1-week-old mice (Figure 8B). The lethal virulence was invariably associated with a sharp drop in the body weight of infected mice that began ~3 days prior to death, in conjunction with clinical signs. It began with decreased activity, ruffled fur, and hunched posture, and often progressed to tremors and hind limb paralysis. Various viral loads were detected postmortem in the brains of all mice that died (8.0 × 10^3^–3.9 × 10^8^ PFU/brain). Non-lethal virulence, in contrast, was characterized by an initial weight loss of various degrees, albeit without obvious clinical signs, and a subsequent recovery, to some extent, that was not complete. At the end of the study, no infectious ZIKV was detected in the brains of any of the mice that survived. In both the lethal and non-lethal virulent cases, no changes in body temperature were observed. Altogether, we found that in CD-1 mice, the three ZIKVs had a wide range of virulence, depending on the virus strain, mouse age, and route of infection.

### 3.6. High Degree of Variation in Interferon (IFN) Sensitivity among Three Molecularly Cloned ZIKVs in Mice Lacking Type I (IFNAR^−/−^) or Both Type I and II IFN (IFNAR^−/−^/IFNGR^−/−^) Receptors

**Figure 9:**
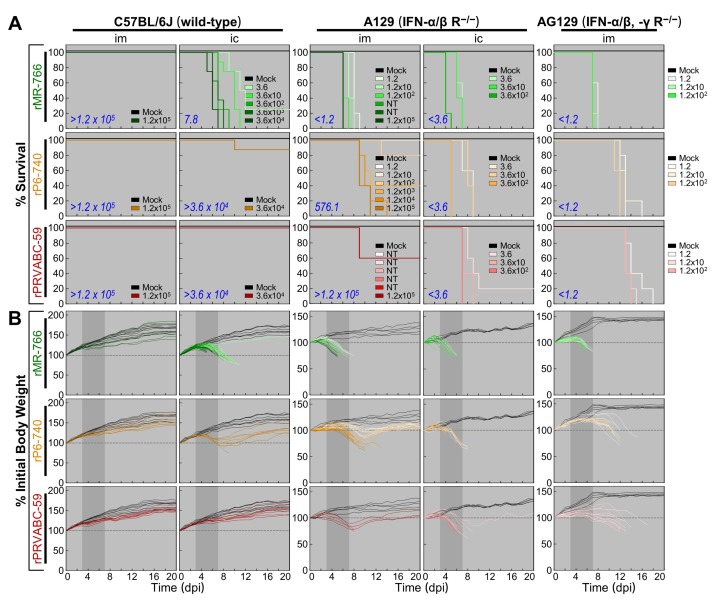
Three molecularly cloned ZIKVs show a full spectrum of variation in IFN sensitivity in mice lacking type I or both type I and II IFN receptors. Groups of 4-week-old C57BL/6J (*n* = 8), A129 (*n* = 5), or AG129 (*n* = 5) mice, approximately half of each sex, were mock-inoculated or inoculated through the intramuscular (im) or intracerebral (ic) route with a maximum dose of 3.6 × 10^4^ or 1.2 × 10^5^ PFU, or serial 10-fold dilutions of rMR-766, rP6-740, or rPRVABC-59. (**A**) Survival curves were created by the Kaplan–Meier method, and LD_50_ values were calculated by the Reed–Muench method and are given in the bottom left corner of each curve; (**B**) Weight changes are plotted, with each mouse indicated by one color-coded line. NT, not tested; dpi, days post-infection.

To compare the contributions of the host IFN response to the virulence of rMR-766, rP6-740, and rPRVABC-59, we examined their neuroinvasiveness and neurovirulence by using groups of 4-week-old A129 (IFNAR^−/−^) mice and groups of age-matched wild-type inbred C57BL/6J mice as a control (Figure 9A). In the control mice, rMR-766 was non-neuroinvasive (im LD_50_, >1.2 × 10^5^ PFU) but neurovirulent (ic LD_50_, 7.8 PFU). In contrast, both rP6-740 and rPRVABC-59 were non-neuroinvasive (im LD_50_, >1.2 × 10^5^ PFU) as well as non-neurovirulent (ic LD_50_, >3.6 × 10^4^ PFU), in agreement with the data obtained in age-matched outbred CD-1 mice (Figure 8A). In A129 mice, however, the neurovirulence of all three ZIKVs was increased sharply, and they became highly neurovirulent (ic LD_50_, <3.6 PFU), with median survival times estimated to be 4 (rMR-766), 5 (rP6-740), and 7 (rPRVABC-59) days, with a lethal dose of 3.6 × 10^2^ PFU/mouse. Similarly, the neuroinvasiveness of the three ZIKVs was also elevated but to different degrees, as evidenced by the estimated im LD_50_ of <1.2 (rMR-766), 576.1 (rP6-740), and >1.2 × 10^5^ (rPRVABC-59) PFU. Noticeably, rPRVABC-59 was nearly non-neuroinvasive in A129 mice. This finding prompted us to further test the neuroinvasiveness of rPRVABC-59, as compared to that of the other two ZIKVs, in 4-week-old AG129 (IFNAR^−/−^/IFNGR^−/−^) mice (Figure 9A). In AG129 mice, all three ZIKVs were highly neuroinvasive (im LD_50_, <1.2 PFU), although the median survival times for the three viruses varied from 7 (rMR-766) to 12 (rP6-740) and 13 (rPRVABC-59) days, with a lethal dose of 1.2 × 10^2^ PFU/mouse. Furthermore, in all three mouse strains (C57BL/6J, A129, and AG129), the two LD_50_-based neuropathogenic properties of the three ZIKVs were always corroborated by the decreases in body weight (Figure 9B), accompanied by the typical clinical signs seen in CD-1 mice. In all the mice that died, various viral loads were detected in their brains postmortem, with higher loads being found in the absence of IFN signaling, i.e., 4.7 × 10^4^–2.0 × 10^8^ PFU/brain for C57BL/6J, 1.3 × 10^6^–1.0 × 10^9^ PFU/brain for A129, and 8.5 × 10^6^–3.6 × 10^9^ PFU/brain for AG129. In the case of all mice that survived, however, we detected no infectious ZIKV in the brain at the end of the study. Taken together, these data show a full range of variation in IFN sensitivity among the three cloned ZIKVs in mice.

## 4. Discussion

Here, we report the first development of three full-length infectious ZIKV cDNAs as BACs for each of three spatiotemporally distinct and genetically divergent ZIKV strains [54]: MR-766 (Uganda, 1947), P6-740 (Malaysia, 1966), and PRVABC-59 (Puerto Rico, 2015). We have also produced 13 ZIKV region-specific polyclonal rabbit antisera capable of identifying all the viral structural and nonstructural proteins and their related species, except for NS2A. Using our functional cDNAs and antibodies in combination with various cell culture and murine model systems, we have demonstrated that the three molecularly cloned cDNA-derived ZIKVs have the nearly same genome-wide viral protein expression profile but differ in their replication kinetics and neuropathogenicity (neuroinvasiveness and neurovirulence), depending on the particular combination of viral and host genetic backgrounds, as well as in the presence or absence of type I/II IFN signaling. In particular, our results demonstrate that type I IFN regulates ZIKV neuroinvasiveness in a virus strain-dependent manner. In all, these reagents offer a new toolbox for viral genome engineering and protein analysis. Together with a roster of in vitro and in vivo infection models, these tools will not only provide an ideal platform for defining the viral and host genetic factors that contribute to ZIKV replication and pathogenesis at the cellular and organismic levels, but also offer promising new avenues for developing and testing an effective, critically needed vaccine against ZIKV.

The advent of functional cDNA-based reverse genetics has revamped the field of RNA viruses [68]. For flaviviruses, however, the cloned cDNAs are commonly unstable because of the toxicity of their prM-E genes in host cells, posing a major technical challenge to functional cDNA construction [69]. In the present study, we cloned a complete cDNA copy of the ZIKV genomic RNA into a BAC vector that is capable of stably housing a DNA fragment of >300 kb in bacteria [70], as we have already done for JEV [53,58]. In the case of all three ZIKVs (MR-766, P6-740, and PRVABC-59), we showed that the structural and functional integrity of their full-length BACs remained stable for at least 80 generations of growth in *E. coli*. To date, the BAC cloning technology has been applied to constructing full-length infectious cDNAs for ~10 members of three plus-strand RNA virus families (*Flaviviridae*, *Arteriviridae*, and *Coronaviridae*), all of which have a large genome size of 11–31 kb [52]. Moreover, we performed site-directed mutagenesis to introduce a point mutation(s) into the full-length infectious cDNA BAC clone of ZIKV P6-740, which demonstrated (i) the functional importance of His-713 within the conserved structural motif E region in the RNA-dependent RNA polymerase domain of ZIKV NS5 for viral RNA replication and (ii) the functional significance of the *N*-glycosylation site at Asn-154 in the viral E protein for its biogenesis. These results indicate that targeted mutations can be engineered by manipulating the infectious ZIKV BACs in *E. coli*. Thus, our BAC-based reverse genetics for ZIKV will facilitate genetic studies of both viral RNA elements and gene products associated with all aspects of ZIKV biology.

We formulated a strategy to assemble three full-length infectious ZIKV cDNAs, each capable of generating m^7^G-capped in vitro-transcribed RNAs identical in nucleotide sequence to their respective genomic RNAs, particularly regarding the 5′- and 3′-end sequences. On the 5′ side, we positioned an SP6 promoter sequence (5′-ATTTAGGGGACACTATAG, with transcription starting at the underlined G) upstream of the first adenine nucleotide of the viral genome to incorporate the dinucleotide cap analog m^7^GpppA in SP6 RNA polymerase-driven in vitro transcription reactions. The importance of an m^7^G cap at the 5′-end of transcribed RNAs in maximizing RNA infectivity was shown by our finding that uncapped RNAs derived from each of the three functional ZIKV cDNAs always had an infectivity >3-logs lower than that of their m^7^G-capped counterparts. On the 3′ side, we placed a unique restriction endonuclease recognition site, *Psr*I [(N_7_↓N_12_)GAACN_6_TAC(N_12_↓N_7_)] or *Bar*I [(N_7_↓N_12_)GAAGN_6_TAC(N_12_↓N_7_)], downstream of the last thymine nucleotide of the viral genome. The use of *Psr*I/*Bar*I for cDNA linearization is particularly advantageous because both are extremely rare-cutting endonucleases that cut out their recognition sequences after any nucleotide, which makes this approach applicable for all plus-strand RNA viruses, regardless of the identity of the nucleotide at the 3′ end of the viral genome. We found that RNA transcripts with 11 ZIKV-unrelated nucleotides hanging on their 3′ ends were ~1-log less infectious than those with authentic 3′ ends, indicating the importance of the authentic 3′ end for the production of infectious ZIKV RNAs.

Several functional cDNAs for ZIKV have hitherto been made using two different strategies, depending on the vector adopted to clone its full-length cDNA and the method applied to create the viral 5′ and 3′ ends: (i) The low-copy plasmid pACYC177 (~15 copies/cell) has been utilized to house a complete cDNA flanked by a 5′ bacteriophage T7 promoter and a 3′ hepatitis delta virus ribozyme (HDVr). This T7-HDVr system, analogous to our SP6-*Psr*I/*Bar*I system, requires an in vitro transcription and transfection of transcribed RNAs into cells for virus recovery. This “RNA-initiated” approach has been implemented to clone the viral genomic RNA of the 2010 Cambodian FSS13025 strain [71]. To circumvent the need for a single plasmid containing a full-length cDNA, in vitro ligation of two or four cDNA fragments pre-cloned individually into the low-copy pACYC177 or high-copy pUC57 (500–700 copies/cell) plasmid, although relatively inefficient, has been done to generate a full-length cDNA template prior to in vitro transcription using the T7-HDVr system for the Ugandan MR-766 (1947), French Polynesian H/PF/2013 (2013), Puerto Rican PRVABC-59 (2015), and Brazilian SPH2015 (2015) and BeH819015 (2015) strains [49,72]. (ii) The low-copy pACNR1811 (10–20 copies/cell) or high-copy pcDNA6.2 (500–700 copies/cell) plasmid is used to house a full-length cDNA containing one or two artificial introns to restrict its instability during propagation in *E. coli*. In this case, a eukaryotic RNA polymerase (RNAP) II-dependent cytomegalovirus (CMV) promoter is positioned before the viral 5′ end, and a pair of HDVr and an SV40 poly(A) signal/RNAP II terminator are placed after the viral 3′ end. Unlike our SP6-*Psr*I/*Bar*I system, the CMV-HDVr system requires transfection of cells with a plasmid carrying the intron-bearing full-length cDNA. This “DNA-initiated” approach has been applied to clone the viral genomic RNA of the Ugandan MR-766 (1947) and Brazilian Paraiba (2015) strains [73,74]. Alternatively, a circular form of the intronless full-length cDNA for the 2015 Brazilian Natal strain has been generated by PCR-mediated joining of eight overlapping cDNA fragments that are pre-cloned individually into the high-copy pUC plasmid [75]. Although far less efficient, a similar PCR-based method has also been reported that uses three overlapping cDNA fragments covering the viral genomic RNA with no joining of these fragments into a circular cDNA [76]. In the present study, we developed a single plasmid-based RNA-initiated reverse genetics system for ZIKV that not only maximizes the genetic stability of its cloned cDNA using a single-copy BAC as a vector, but also optimizes the synthesis of infectious RNAs in vitro using the SP6-*Psr*I/*Bar*I system.

ZIKV circulates in a sylvatic cycle between nonhuman primates (NHPs) and forest-dwelling mosquitoes, as well as in an urban cycle between humans and town-dwelling mosquitoes [12]. Apart from NHPs, however, information is scarce on any potential animal hosts or reservoirs for ZIKV transmission. Using our three cDNA-derived genetically distinct ZIKVs, we evaluated their ability to infect and replicate in 17 animal cell lines from 12 different species (monkeys, humans, mosquitoes, mice, cows, pigs, sheep, goats, horses, dogs, cats, and chickens). Our data showed that ZIKV has a broad cell tropism in vitro, being capable of establishing productive infection in 16 of the 17 cell lines we tested, although its growth rate and ability to induce CPE varied widely depending on both the specific virus strain and host cell line. Of particular note, all three ZIKVs grew readily in both porcine ST and equine NBL-6 cells, with their growth kinetics similar to those observed in simian Vero cells; generally, they also replicated and spread equally well in ovine SFF-6 cells and in aedine C6/36 cells. These results raise a question as to whether several agriculturally important domestic animals (e.g., pigs, sheep, and horses) are susceptible to ZIKV infection, particularly when young. With respect to this question, two recent studies have reported that fetal and neonatal piglets are susceptible to experimental infection with ZIKV [77,78]. Another pilot study has suggested that a range of adult animals, including rabbits, goats, pigs, cattle, chickens, and ducks, are less likely to act as animal hosts for ZIKV, based on the levels of viremia and neutralizing antibody titer induced following ZIKV infection; of these animals, however, rabbits and pigs are proposed to be able to serve as sentinels for ZIKV surveillance [79]. Clearly, further investigation is needed to elucidate the susceptibility of these animals to ZIKV infection, preferentially in the presence or absence of type I IFN signaling, and might provide new opportunities to increase our understanding of ZIKV biology and to develop a non-murine model for ZIKV research. Moreover, we discovered a nonsusceptible bovine MDBK cell line that has a block in ZIKV entry but fully supports the subsequent post-entry steps; however, this cell line remained highly susceptible to infection by two other enveloped RNA viruses, BVDV [80] and VSV [81], which like ZIKV [63], enter the cells through clathrin-dependent endocytic pathway. This ZIKV-nonsusceptible bovine cell line thus offers a unique opportunity to identify the host factors involved in ZIKV entry.

To our knowledge, this work is the first to generate such a large panel of 13 ZIKV region-specific antibodies that can identify experimentally nearly all the viral gene products and their related species in ZIKV-infected Vero cells and define all three structural proteins associated with extracellular virions. Our data are in overall good agreement with the current model for flavivirus polyprotein processing [21], but they have also revealed a considerable number of previously undescribed, presumed cleavage intermediates or further cleavage/degradation products. The main findings from our study are as follows: (1) While the full-length 13 kDa C and its one or two processed 10 to 11 kDa proteins were accumulated intracellularly, the extracellular virion-associated C’ protein appeared as a tightly spaced 12 kDa doublet. (2) For each of the two viral surface glycoproteins (24 kDa prM and 54/56 kDa E), two or three smaller products were also cell-associated but not virion-associated. (3) Only the 45 kDa NS1, and not its theoretically frameshift-derived product NS1’, was expressed. (4) In addition to the intact 14 kDa NS2B, its processed 11 kDa product was also stained, although weakly. (5) The full-length 69 kDa NS3 was processed to yield multiple truncated species of 33–60 kDa, of which the C-terminal 34 kDa fragment was the most prominent species. (6) The predicted 16 kDa NS4A was completely undetectable, but three unpredicted NS4A-related proteins were readily identified, i.e., a major doublet at 14 kDa (NS4A’) and two minor protein clusters at 29 kDa (NS4A^p29^) and 35 kDa (NS4AB^p35^). (7) Not only the predicted 27 kDa NS4B but also two unpredicted NS4B-related proteins were observed, one at 11 kDa (NS4B^p11^) and the other at 35 kDa (NS4AB^p35^). However, the functional importance of the previously undescribed cleavage intermediates or further cleavage/degradation products in ZIKV biology remains to be determined.

Much progress has been made over the past year in developing animal models (i.e., mice and NHPs) for ZIKV [43]. To date, the mouse is the most feasible small animal that mimics aspects of ZIKV infection in humans, albeit with some limitations resulting from species differences in innate immunity, reproductive system, and fetal development. Previously, no productive infection was detected when several strains of immunocompetent adult mice were inoculated peripherally with diverse ZIKVs, but robust peripheral ZIKV infection causing substantial morbidity and mortality was observed in both immunocompromised adult and immunocompetent neonatal mice [44,46,82,83,84,85,86,87,88,89]. We call attention, however, to the large variation in ZIKV pathogenicity among the previous studies, which were conducted by inoculating a variety of ZIKVs into different strains of mice via various routes. In the current report, we have shown in immunocompetent CD-1 mice at 1, 2, and 4 weeks of age that ZIKV neuropathogenicity can only be defined in the context of a virus-host combination, particularly depending on both viral strain and mouse age, as evidenced by comparison of the neuroinvasiveness and neurovirulence of our three molecularly cloned, genetically distinct ZIKVs: (i) rMR-766 exhibited neonate-specific age-dependent neuroinvasiveness but displayed a high level of neurovirulence at all three ages. (ii) rP6-740 had little-to-no neuroinvasiveness at all three ages but possessed neonate-specific age-dependent neurovirulence. (iii) rPRVABC-59 was non-neuroinvasive and non-neurovirulent at all three ages. Also, we showed marked differences in IFN sensitivity among the three ZIKVs: In 4-week-old A129 (IFNAR^−/−^) mice, the three ZIKVs were uniformly neurovirulent but varied in neuroinvasiveness (rMR-766, neuroinvasive; rP6-740, intermediate; and rPRVABC-59, almost non-neuroinvasive); however, all three ZIKVs, including rPRVABC-59, were neuroinvasive in age-matched AG129 (IFNAR^−/−^/IFNGR^−/−^) mice. Consistent with previous work, we noted a greater susceptibility and more severe disease in AG129 mice than in A129 mice [44,45,46,47,48,49]. In all fatal cases, the mortality was related to the productive infection in the brain, coupled with tremors, ataxia, and hind limb paralysis.

In conclusion, use of our newly developed comparative functional genomics and immunologic tools, combined with various cell culture and mouse infection model systems, will facilitate further research leading to ZIKV disease prevention and therapy, as well as an in-depth understanding of ZIKV biology.

## Figures and Tables

**Table 1:** Cells used in this study.

Organism	Cell	Tissue	Growth Medium *^a^*	Culture Condition	Source (Catalog Number) *^b^*
Human	HEK	Embryo, kidney	MEM supplemented with 10% FBS, 2 mM L-glutamine, 0.1 mM NEAA, 1.0 mM SP, and PS	37^o^C, 5% CO_2_	ATCC (CRL-1573)
Human	Huh-7	Liver	DMEM supplemented with 10% FBS, 0.1 mM NEAA, and PS	37^o^C, 5% CO_2_	Charles M. Rice, RU
Human	SH-SY5Y	Bone marrow	A 1:1 mixture of MEM and Ham's F-12 nutrient medium supplemented with 10% FBS, 0.1 mM NEAA, and PS	37^o^C, 5% CO_2_	ATCC (CRL-2266)
Mouse	MEF	Embryo (C57BL/6), fibroblast	DMEM supplemented with 10% FBS and PS	37^o^C, 5% CO_2_	ATCC (SCRC-1008)
Mouse	NIH/3T3	Embryo (NIH/Swiss), fibroblast	DMEM supplemented with 10% FBS and PS	37^o^C, 5% CO_2_	ATCC (CRL-1658)
Mouse	NSC-34	Motor neuron-like hybrid	DMEM (without SP) supplemented with 10% FBS and PS	37^o^C, 5% CO_2_	Cedarlane (CLU140)
Monkey	Vero	Kidney	α-MEM supplemented with 10% FBS and PS	37^o^C, 5% CO_2_	ATCC (WHO-Vero)
Cow	BT	Turbinate	DMEM (without SP) supplemented with 10% HS and PS	37^o^C, 5% CO_2_	ATCC (CRL-1390)
Cow	MDBK	Kidney	DMEM (without SP) supplemented with 10% HS and PS	37^o^C, 5% CO_2_	ATCC (CCL-22)
Pig	ST	Testis	α-MEM supplemented with 10% FBS and PS	37^o^C, 5% CO_2_	ATCC (CRL-1746)
Sheep	SFF-6	Fetus, fibroblast	DMEM supplemented with 15% FBS and PS	37^o^C, 5% CO_2_	Irina A. Polejaeva, USU
Goat	GFF-4	Fetus, fibroblast	DMEM supplemented with 15% FBS and PS	37^o^C, 5% CO_2_	Irina A. Polejaeva, USU
Horse	NBL-6	Skin, dermis	EMEM supplemented with 10% FBS and PS	37^o^C, 5% CO_2_	ATCC (CCL-57)
Dog	MDCK	Kidney	MEM supplemented with EBSS, 10% FBS, 0.1 mM NEAA, 1.0 mM SP, and PS	37^o^C, 5% CO_2_	ATCC (CCL-34)
Cat	CRFK	Kidney, cortex	MEM supplemented with EBSS, 10% HS, 0.1 mM NEAA, 1.0 mM SP, and PS	37^o^C, 5% CO_2_	ATCC (CCL-94)
Chicken	CEF	Embryo, fibroblast	DMEM supplemented with 10% FBS and PS	37^o^C, 5% CO_2_	Sung-June Byun, KNIAS
Mosquito	C6/36	Larva (*Aedes albopictus*)	MEM supplemented with EBSS, 10% FBS, 2 mM L-glutamine, 0.1 mM NEAA, 1.0 mM SP, and PS	28^o^C, 5% CO_2_	ATCC (CRL-1660)

*^a^* MEM, minimum essential medium; α-MEM, alpha minimum essential medium; DMEM, Dulbecco’s modified eagle medium; EMEM, Eagle’s minimum essential medium; EBSS, Earle’s balanced salt solution; FBS, fetal bovine serum; HS, horse serum; NEAA, nonessential amino acids; SP, sodium pyruvate; PS, penicillin-streptomycin. *^b^* ATCC, American Type Culture Collection; RU, Rockefeller University; USU, Utah State University; KNIAS, Korea National Institute of Animal Science.

**Table 2:** Oligonucleotides used in this study.

Oligonucleotide	Sequence *^a^* (5’ to 3’)	Position *^b^*	Direction
Z1RT	GCTATTGGGTTCATGCCACAGATGGTCATCA	4531–4561	Reverse
Z1F	tatgtttaaacAGTTGTTGATCTGTGTGAATCAGACTGCGA	1–30	Forward
Z1R	tatggcgcgccAGGACCACCTTGAGTATGATCTCTCTCATG	4502–4531	Reverse
Z2RT	ATTGTCATTGTGTCAATGTCAGTCACCACTA	7369–7399	Reverse
Z2F	tatgtttaaacTCATTGTTTGGAGGAATGTCCTGGTTCTCA	2340–2369	Forward
Z2R	tatggcgcgccTCAATGTCAGTCACCACTATTCCATCCACA	7358–7387	Reverse
Z3RT	CTCCAGTTCAGGCCCCAGATTGAAGGGTGGGG	10603–10634	Reverse
Z3F	tatgtttaaacGGAAGTCCCAGAGAGAGCCTGGAGCTCAGG	5627–5656	Forward
Z3R	tatggcgcgccAAGGGTGGGGAAGGTCGCCACCTTCTTTTC	10583–10612	Reverse
S123-5sp1F	ctaggatccttaattaacctgcagggggctgtta		Forward
S123-5sp1R	GATCAACAACTctatagtgtcccctaaatc	1–11	Reverse
S1-5sp2F	ggacactatagAGTTGTTGATCTGTGTGAGTC	1–21	Forward
S1-5sp2R	tatccgcggTAGCGCAAACCCGGGGTTCCTGAAT	860–884	Reverse
S1-3roF	tatccgcggGGAAAAAGGGAGGACTTATGGTGTG	10191–10215	Forward
S1-3roR	agggcggccgcgtatgtcgcgttccgtacgttctagAGAAACCATGGATTTCCCCACACC	10785–10807	Reverse
S23-5sp2F	ggacactatagAGTTGTTGATCTGTGTGAATC	1–21	Forward
S23-5sp2R	tatccgcggAACGCAAAGCCAGGGTTCCTGAATA	859–883	Reverse
S23-3roF	tatccgcggGGGAAAAAGGGAAGACTTATGGTGT	10190–10214	Forward
S23-3roR	agggcggccgcgtatgtcgccttccgtacgttctagAGACCCATGGATTTCCCCACACCG	10784–10807	Reverse
ZikaC-F	tttgaattcGGTCTCATCAATAGATGGGGT	297–317	Forward
ZikaC-R	tttctcgagctattaTCGTCTCTTCTTCTCCTTCCT	399–419	Reverse
ZikaM-F	tttgaattcGCTGTGACGCTCCCCTCCCAT	753–773	Forward
ZikaM-R	tttctcgagctattaGACTCTAATCAAGTGCTTTGT	828–848	Reverse
ZikaE-F	tttgaattcCAGCACAGTGGGATGATCGTT	1416–1436	Forward
ZikaE-R	tttctcgagctattaTCCTAGGCTTCCAAAACCCCC	1518–1538	Reverse
ZikaNS4A-F	tttgaattcGGAGCGGCTTTTGGAGTGATG	6465–6485	Forward
ZikaNS4A-R	tttctcgagctattaGGTCTCCGGCAATTGGGCCGC	6597–6617	Reverse
ZikaNS4B-F	tttgaattcGTGACTGACATTGACACAATG	7374–7394	Forward
ZikaNS4B-R	tttctcgagctattaGGAAGTTGCGGCTGTGATCAG	7506–7526	Reverse
ZikaF	GAAGTGGAAGTCCCAGAGAG	5622–5641	Forward
ZikaR	TGCTGAGCTGTATGACCCG	5757–5775	Reverse
ZikaProbe	FAM-TGGAGCTCAGGCTTTGATTGGGTGAC-BHQ1	5646–5671	Forward
VeroF	*AGCGGGAAATCGTGCGTGAC*	624–643	Forward
VeroR	*CAATGGTGATGACCTGGCCA*	742–761	Reverse
VeroProbe	HEX-*CACGGCGGCTTCTAGCTCCTCCC*-BHQ2	694–716	Forward

*^a^* ZIKV-specific sequences are indicated in uppercase normal letters, and Vero β-actin-specific sequences are shown in uppercase italic letters. Other nonviral sequences are indicated in lowercase letters. Restriction enzyme sites used for cDNA cloning are underlined. FAM, 6-Carboxyfluorescein; HEX, Hexachlorofluorescein; BHQ, Black hole quencher. *^b^* Nucleotide position refers to the complete genome sequence of ZIKV PRVABC-59 (GenBank accession number KX377337) or to the mRNA sequence of Vero β-actin (GenBank accession number AB004047).

## Supplementary Materials

The following are available online at http://www.mdpi.com/1999-4915/10/8/422/s1, Figure S1: A single C^9804^→U substitution essentially eliminates the specific infectivity of RNA transcripts derived from a full-length infectious cDNA clone of ZIKV P6-740, Figure S2: Three functional ZIKV cDNA clones are stably propagated in bacteria as BACs, Figure S3: ZIKV growth kinetics and cytopathogenicity in cell cultures depend on the particular combination of virus strain and host cells, Figure S4: MDBK cells are highly susceptible to infection with both BVDV and VSV, Figure S5: Details of the 15 JEV region-specific rabbit antisera used to detect their antigenically cross-reactive ZIKV counterparts, Figure S6: Details of the seven ZIKV region-specific rabbit antisera used to identify ZIKV gene products and their related species, Figure S7: A missense mutation eliminating the *N*-glycosylation site at Asn-154 in the viral protein E of rP6-740 is responsible for the observed lower molecular weights of the E and its two related proteins, Figure S8: Multiple NS4A- and NS4B-related proteins are accumulated in ZIKV-infected cells.
