# Supplementary material for: Functional Genomics and Immunologic Tools: The Impact of Viral and Host Genetic Variations on the Outcome of Zika Virus Infection
Source: Viruses. 2018 Aug 11;10(8):422. doi: 10.3390/v10080422 (PMC6116225; doi:10.3390/v10080422)
Supplement: Supplementary file 1 [file viruses-10-00422-s001.zip › viruses-329375-supplementary.docx]

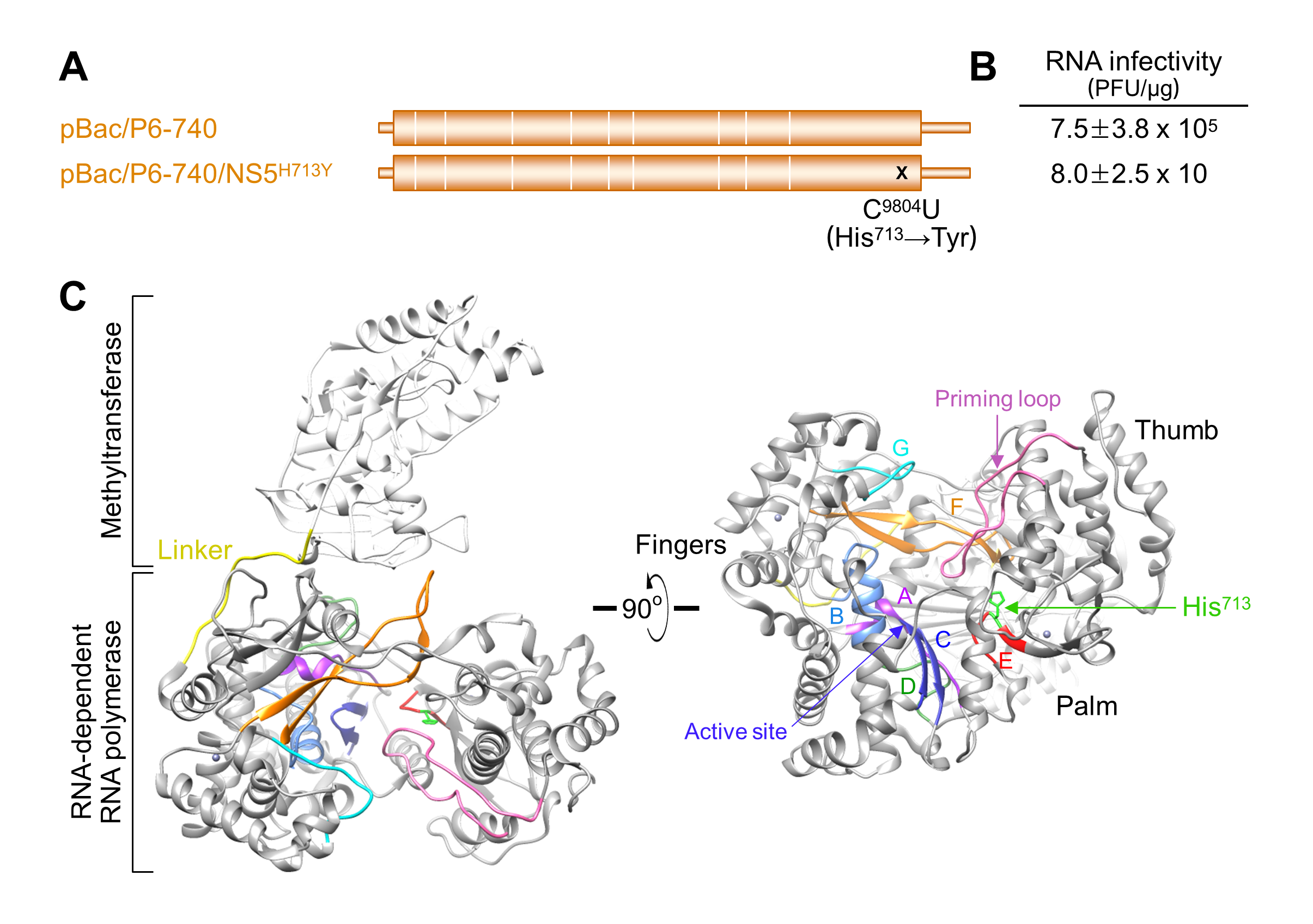


**Figure S1.** A single C^9804^→U substitution essentially eliminates the specific infectivity of RNA transcripts derived from a full-length infectious cDNA clone of ZIKV P6-740. (**A**) Schematic representation showing the locus of the C^9804^→U substitution (pBac/P6-740/NS5^H713Y^) replacing a His with Tyr at position 713 of the viral NS5 protein in the context of the full-length infectious cDNA clone of ZIKV P6-740 (pBac/P6-740). (**B**) RNA infectivity. After linearization with *Bar*I, each full-length cDNA was used as a template for *in vitro* run-off transcription with SP6 RNA polymerase in the presence of the dinucleotide cap analog m^7^GpppA. Capped RNA transcripts were then transfected into Vero cells to determine the number of infectious centers (plaques) counterstained with crystal violet at 5 days after transfection. Means and standard deviations from two independent experiments are shown. (**C**) Mapping of His^713^ on the crystal structure of ZIKV NS5. Ribbon representation showing the arrangement of the methyltransferase and RNA-dependent RNA polymerase domains of ZIKV NS5 (PDB accession code 5U0B). Highlighted in color around the catalytic active site of the RNA-dependent RNA polymerase domain are the seven structural motifs (A to G), priming loop, and His^713^ residue.


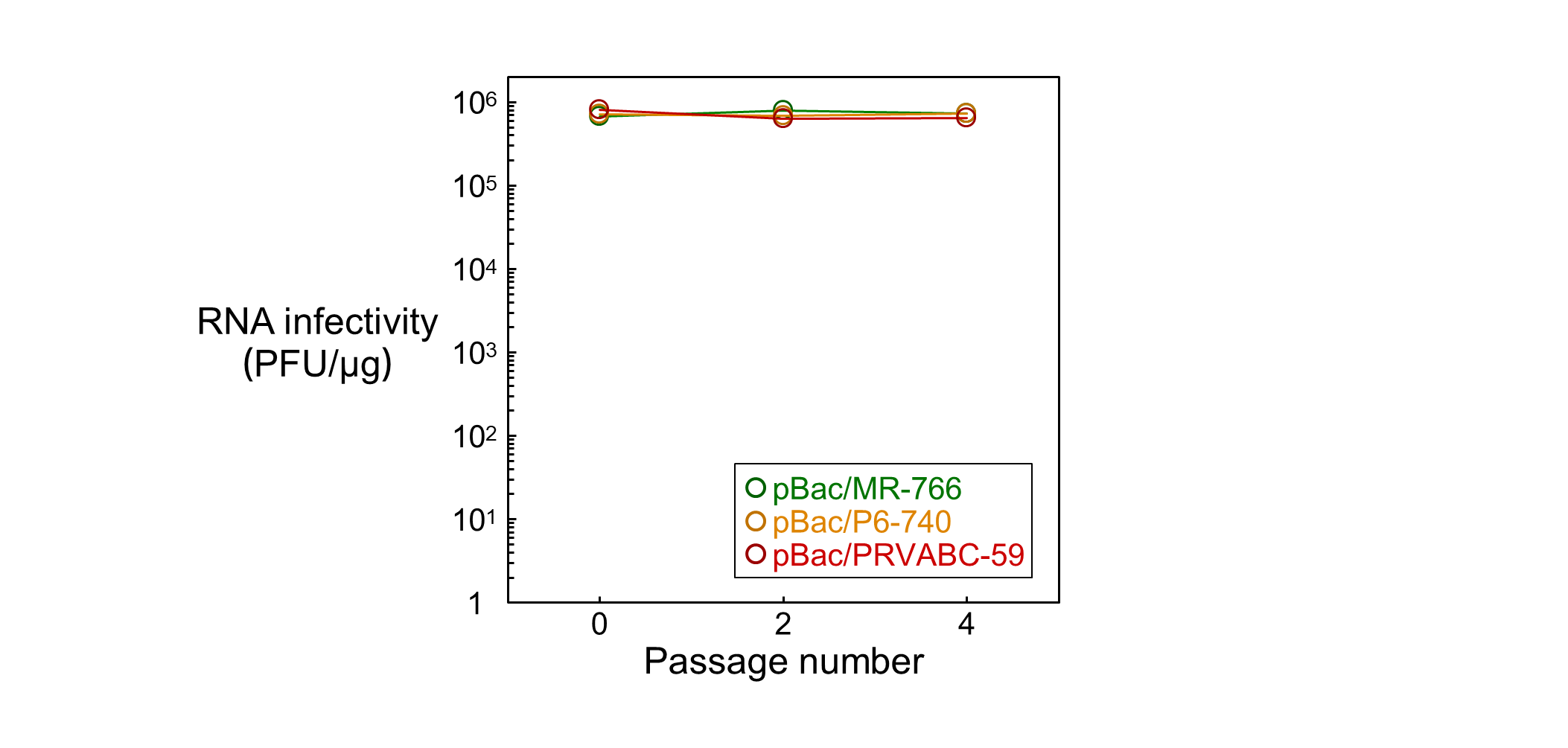


**Figure S2.** Three functional ZIKV cDNA clones are stably propagated in bacteria as BACs. A colony of *E. coli* DH10B cells transformed with each of three functional ZIKV BACs (pBac/MR-766, pBac/P6-740, and pBac/PRVABC-59) was picked randomly and grown at 35^o^C overnight in 2xYT medium with chloramphenicol (passage 0). Cells from these initial cultures were then passaged four times by diluting them 10^6^-fold daily. At passages 0, 2, and 4, the ZIKV BACs were purified, linearized, and transcribed *in vitro* for the synthesis of capped RNAs. The transcribed RNAs were subsequently transfected into Vero cells to determine their specific infectivity.


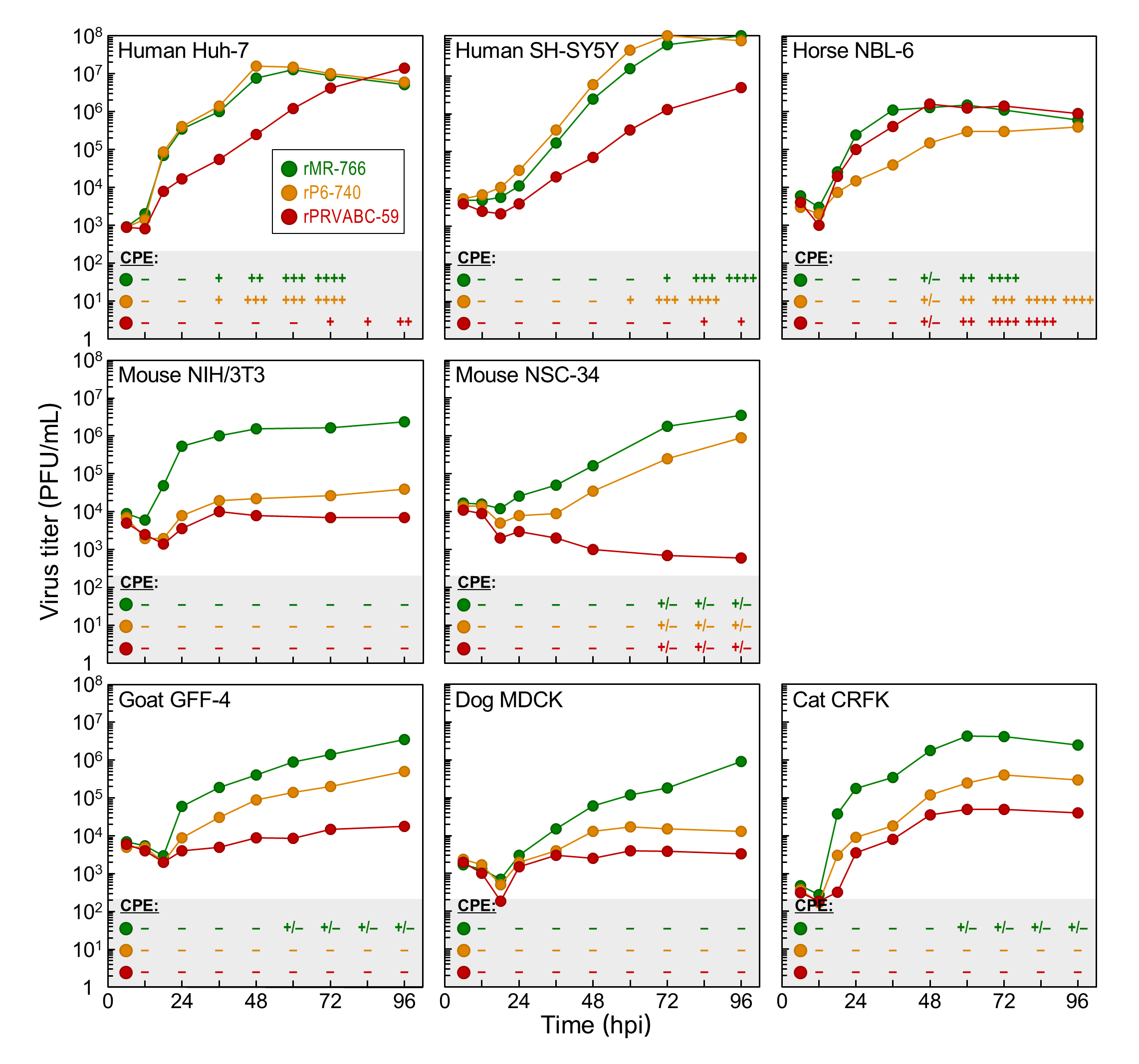


**Figure S3.** ZIKV growth kinetics and cytopathogenicity in cell cultures depend on the particular combination of virus strain and host cells. Each of three molecularly cloned ZIKVs (rMR-766, rP6-740, and rPRVABC-59) was used to infect the indicated cells at an MOI of 1. At the time points marked in the figure, cells were examined microscopically to determine the degrees of ZIKV-induced CPE (–, 0%; +, 0-25%; ++, 25-50%; +++, 50-75%; ++++, 75-100% cell death), and supernatants were harvested to evaluate the levels of virus production by plaque assays on Vero cells. hpi, hours post-infection.


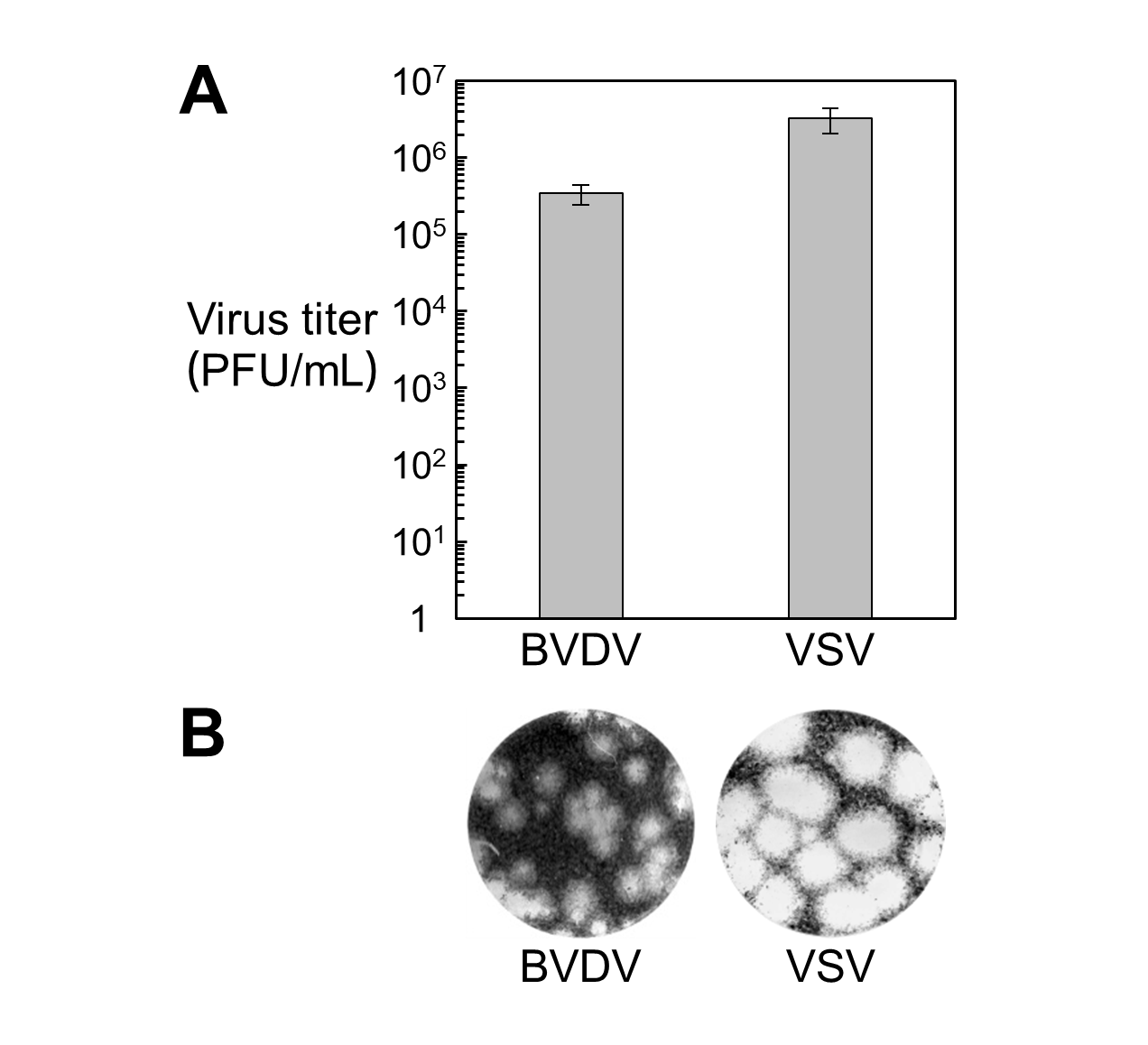


**Figure S4.** MDBK cells are highly susceptible to infection with both BVDV and VSV. MDBK cells were infected at an MOI of 1 with BVDV (strain NADL) or VSV (strain Indiana). (**A**) Viral replication. At 36 hours post-infection, culture supernatants were tested to measure the infectious virus yields by plaque assays on MDBK cells. (**B**) Representative plaques. At 3 days post-infection, cell monolayers maintained under a semisolid overlay medium were counterstained with crystal violet to visualize the infectious plaques.


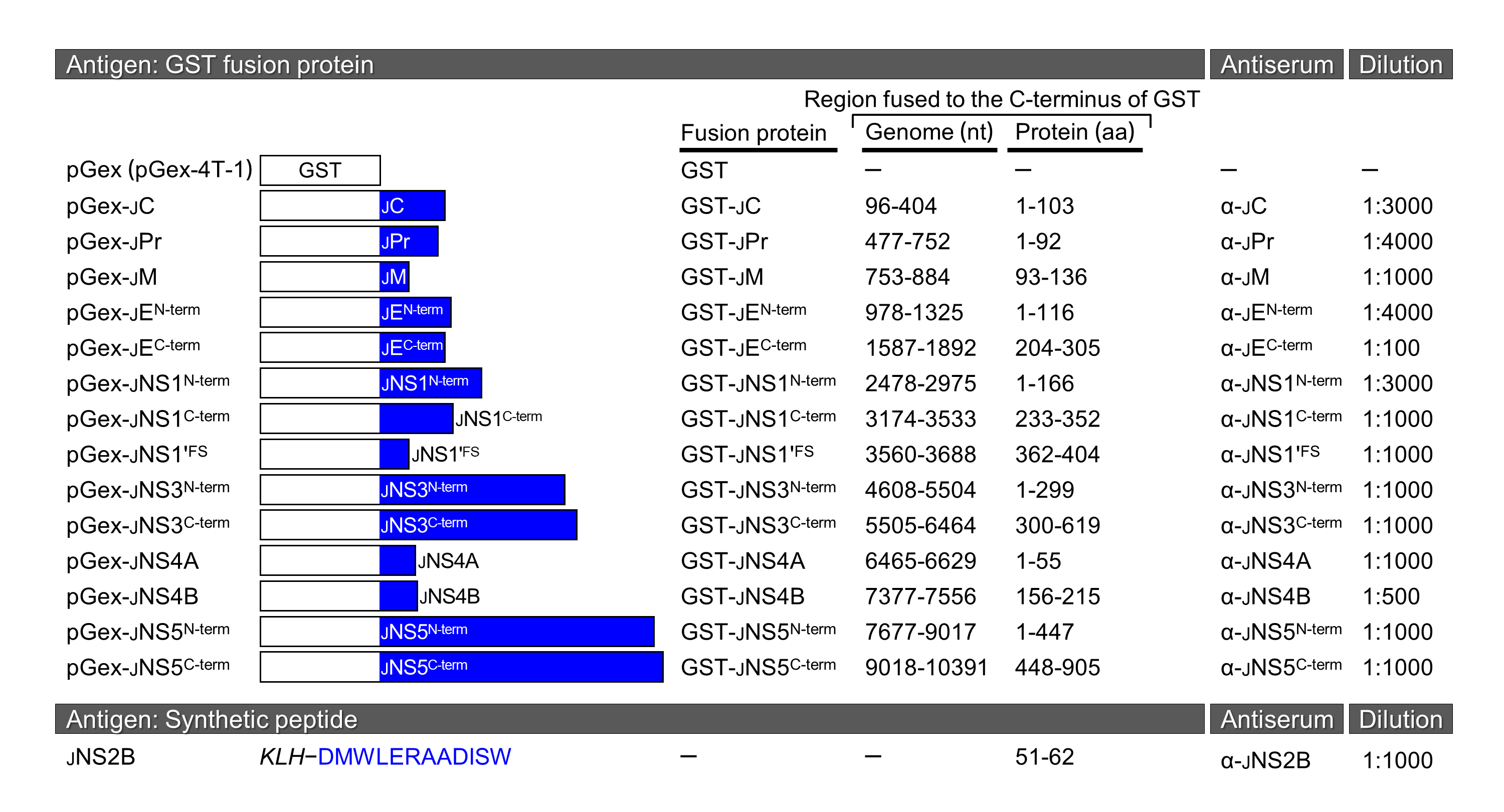


**Figure S5.** Details of the 15 JEV region-specific rabbit antisera used to detect their antigenically cross-reactive ZIKV counterparts. A collection of 15 rabbit antisera covering nearly all parts of the JEV protein-coding regions were raised by immunization with 14 *E. coli*-expressed glutathione-S-transferase (GST) fusion proteins (α-JC, α-JPr, α-JM, α-JE^N-term^, α-JE^C-term^, α-JNS1^N-term^, α-JNS1^C-term^, α-JNS1'^FS^, α-JNS3^N-term^, α-JNS3^C-term^, α-JNS4A, α-JNS4B, α-JNS5^N-term^, and α-JNS5^C-term^) or with a keyhole limpet hemocyanin (KLH)-conjugated synthetic oligopeptide (α-JNS2B). The nucleotide (nt) and amino acid (aa) positions of the viral antigenic regions (blue) are based on the complete genomic sequence of JEV SA_14_ (GenBank accession no. KU323483; see also Figure 5A). The working dilutions of the rabbit antisera used in our study are given.


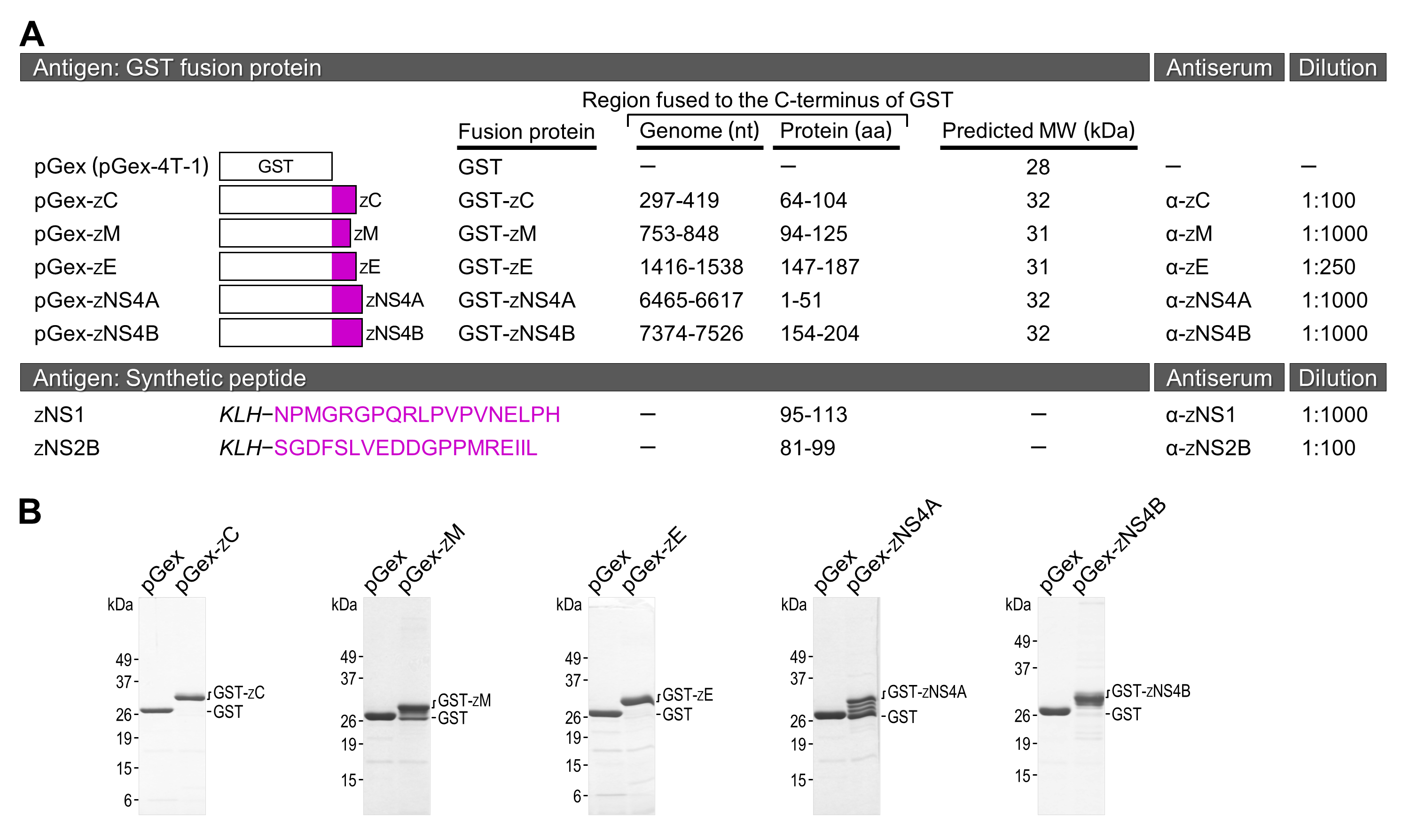


**Figure S6.** Details of the seven ZIKV region-specific rabbit antisera used to identify ZIKV gene products and their related species. (**A**) A panel of seven rabbit antisera, each recognizing a 19- to 51-aa region defined in the ZIKV protein-coding sequences, were generated using five *E. coli*-expressed glutathione-S-transferase (GST) fusion proteins (α-ZC, α-ZM, α-ZE, α-ZNS4A, and α-ZNS4B) or two keyhole limpet hemocyanin (KLH)-conjugated synthetic oligopeptides (α-ZNS1 and α-ZNS2B) as antigens. The nucleotide (nt) and amino acid (aa) positions of the viral antigenic regions (magenta) are based on the complete genomic sequence of ZIKV PRVABC-59 (GenBank accession no. KX377337; see also Figure 6A). The working dilutions of the rabbit antisera used in this study are presented. (**B**) Production of five GST-tagged recombinant proteins. GST fusion proteins were expressed from pGex-4T-1 vector in *E. coli* BL21 and purified from bacterial lysates by affinity chromatography using glutathione-Sepharose. Purified proteins were resolved by SDS-PAGE on a glycine gel and stained with Coomassie blue.


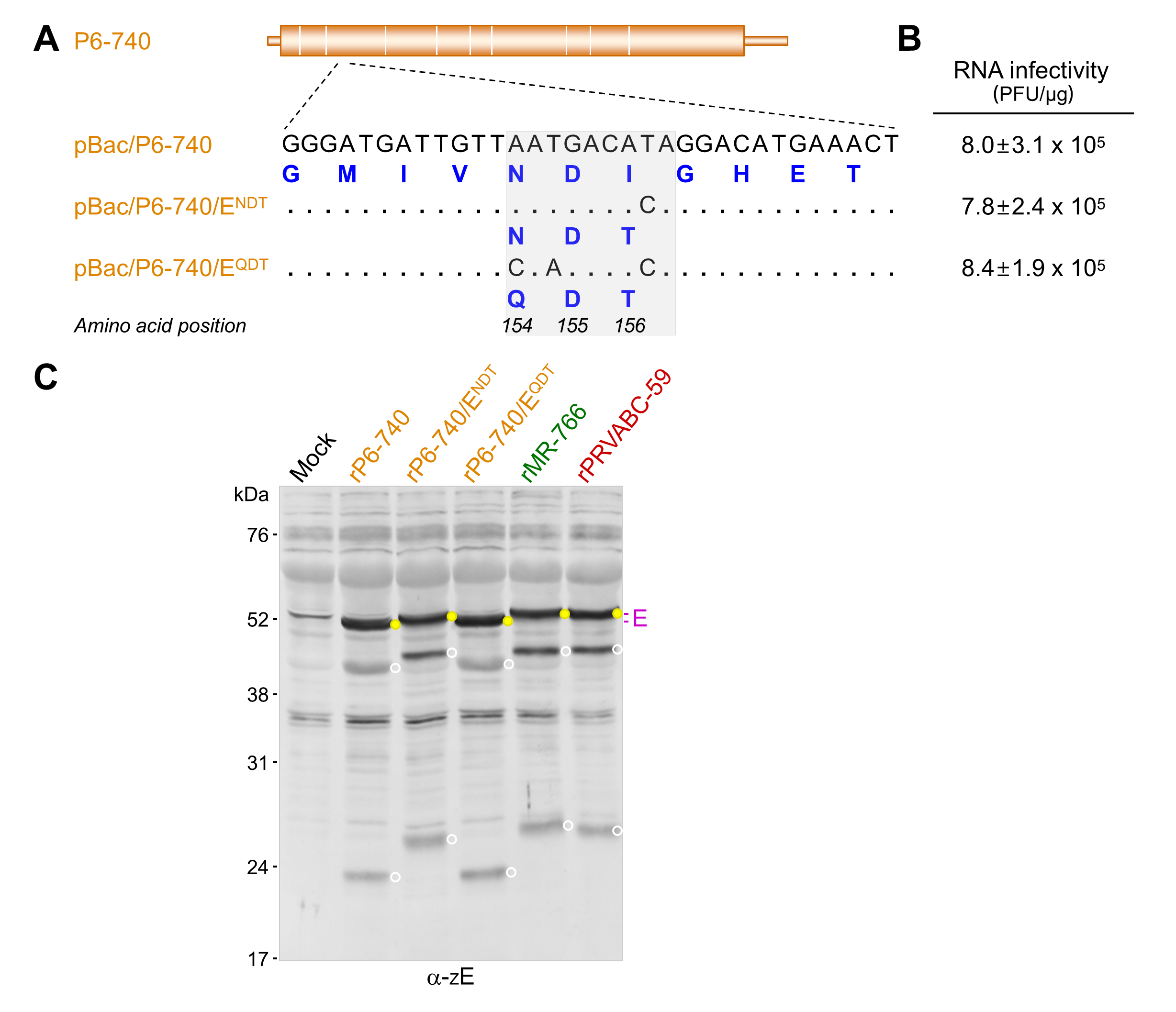


**Figure S7.** A missense mutation eliminating the N-glycosylation site at Asn-154 in the viral protein E of rP6-740 is responsible for the observed lower molecular weights of the E and its two related proteins. (**A**) Schematic representation illustrating the parental full-length cDNA clone (pBac/P6-740) and two E N-glycosylation mutants (pBac/P6-740/E^NDT^ and pBac/P6-740/E^QDT^). The genomic RNA of ZIKV P6-740 is depicted at the top. Below the diagram of the viral genome, the nucleotide and amino acid sequences corresponding to positions 154 to 156 of the parental and its two mutants are shown. Dots indicate identical nucleotides. (**B**) RNA infectivity. Once linearized with *Bar*I, each full-length cDNA was used as a template for *in vitro* run-off transcription with SP6 RNA polymerase in the presence of the dinucleotide cap analog m^7^GpppA. Capped RNA transcripts were then transfected into Vero cells to estimate the number of infectious centers (plaques) that were counterstained with crystal violet at 5 days post-transfection. Means and standard deviations from two independent experiments are shown. (**C**) Identification of viral E protein and its related species in ZIKV-infected cells by immunoblotting. Vero cells were mock-infected or infected at an MOI of 1 with each of three rP6-740 derivatives (rP6-740, rP6-740/E^NDT^, and rP6-740/E^QDT^) and two other ZIKVs (rMR-766 and rPRVABC-59, for comparison). At 20 hours post-infection, total cell lysates were resolved by SDS-PAGE on a glycine gel and analyzed by immunoblotting with α-ZE rabbit antiserum. Molecular size markers are shown on the left side of the blot, and the full-length E (yellow dot) and its two related proteins (white circle) are indicated on the blot.


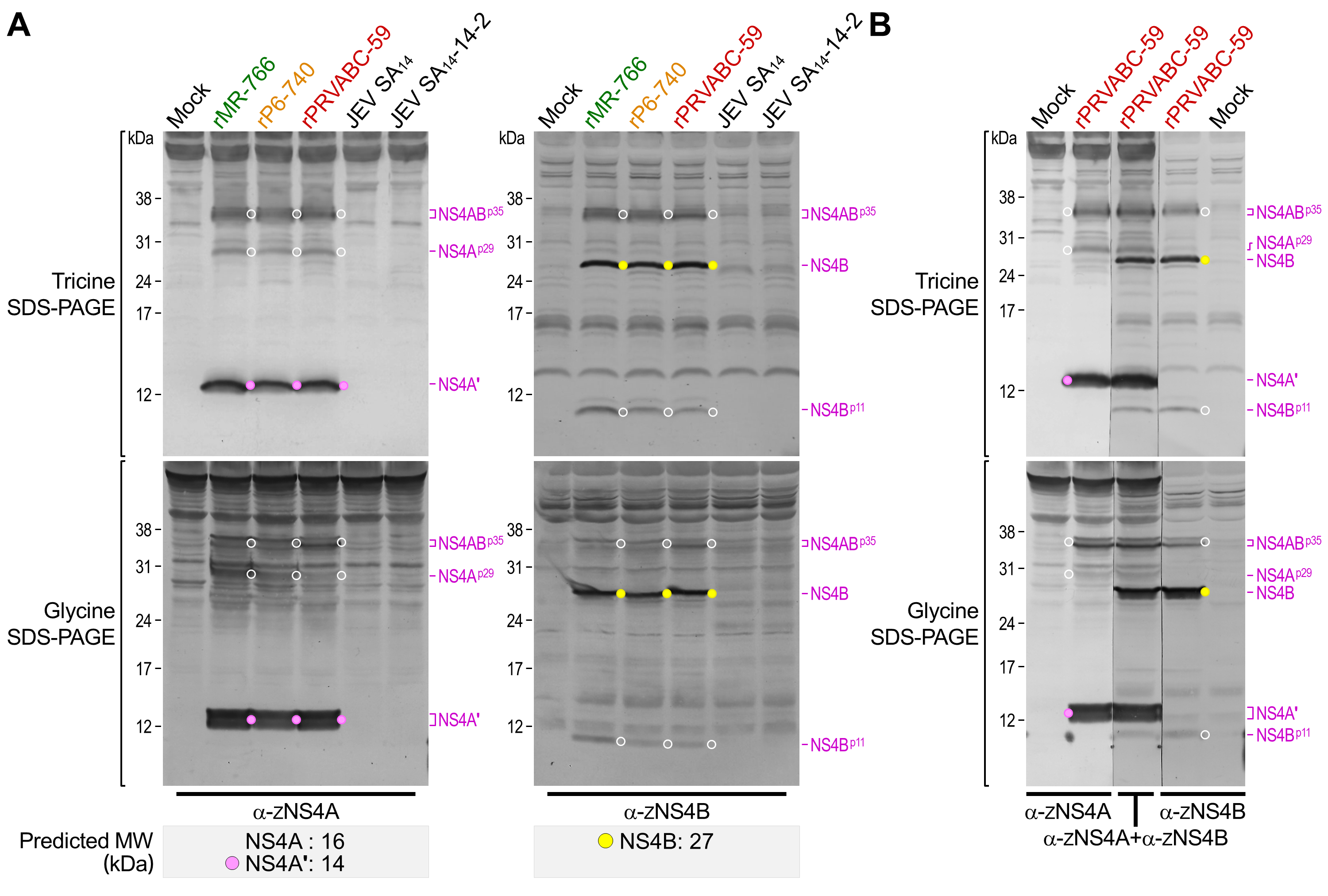


**Figure S8.** Multiple NS4A- and NS4B-related proteins are accumulated in ZIKV-infected cells. Vero cells were mock-infected or infected at an MOI of 1 with each of three ZIKVs (rMR-766, rP6-740, and rPRVABC-59) or two JEVs (SA_14_ and SA_14_-14-2). (**A**) At 20 hours post-infection, total cell lysates were separated by glycine- or tricine-SDS-PAGE and analyzed by immunoblotting with α-ZNS4A or α-ZNS4B. (**B**) Two sets of the same lysates from mock- and rPRVABC-59-infected Vero cells were run side-by-side in a glycine or tricine gel and transferred to a single membrane. The membrane was split into two parts, each stained with either α-ZNS4A or α-ZNS4B. In parallel, an aliquot of the same rPRVABC-59-infected cell lysate was also included in between the two sample sets, and the corresponding membrane strip was probed with a mixture of both α-ZNS4A and α-ZNS4B to serve as a reference for all the immunoreactive proteins. Provided below the blot are the molecular weights of predicted ZIKV NS4A, NS4A’ and NS4B proteins, and marked on the blot are the predicted proteins (yellow or pink dot) and presumed cleavage intermediates or further cleavage/degradation products (white circle).
